# Four species of bacteria deterministically assemble to form a stable biofilm in a millifluidic channel

**DOI:** 10.1038/s41522-021-00233-4

**Published:** 2021-08-05

**Authors:** A. Monmeyran, W. Benyoussef, P. Thomen, N. Dahmane, A. Baliarda, M. Jules, S. Aymerich, N. Henry

**Affiliations:** 1 Sorbonne Université, CNRS, Laboratoire Jean Perrin (UMR 8237), 4 place Jussieu, F-75005 Paris, France; 2 Université Paris-Saclay, INRAE, AgroParisTech, Micalis Institute, 78350 Jouy-en-Josas, France; 3 Present Address: Université Côte d’Azur, CNRS UMR 7010, Institut de Physique de Nice, Parc Valrose, 06108 Nice, France

**Keywords:** Microbial communities, Biofilms

## Abstract

Multispecies microbial adherent communities are widespread in nature and organisms, although the principles of their assembly and development remain unclear. Here, we test the possibility of establishing a simplified but relevant model of multispecies biofilm in a non-invasive laboratory setup for the real-time monitoring of community development. We demonstrate that the four chosen species (*Bacillus thuringiensis*, *Pseudomonas fluorescens*, *Kocuria varians,* and *Rhodocyclus* sp.) form a dynamic community that deterministically reaches its equilibrium after ~30 h of growth. We reveal the emergence of complexity in this simplified community as reported by an increase in spatial heterogeneity and non-monotonic developmental kinetics. Importantly, we find interspecies interactions consisting of competition for resources—particularly oxygen—and both direct and indirect physical interactions. The simplified experimental model opens new avenues to the study of adherent bacterial communities and their behavior in the context of rapid global change.

## Introduction

Microbial communities are essential for global geochemical cycles and ecological equilibria^1^. In nature, these communities typically comprise hundreds of distinct taxa with an enormous potential for direct and indirect interactions^2^. Such ecosystems are the topic of increasing interest to the scientific community, regarding the possibility of predicting and mitigating their responses to global environmental change^3^. High-throughput sequencing and meta-omics approaches coupled with computational models can provide invaluable insight into the taxonomic and functional structures of complex microbial communities harvested from their natural habitats, e.g., soils or oceans^4–6^. Previous studies have shown that multiple examples of ecological interactions, such as syntrophy^7^, catabolic parasitism^8^, or competition for nutrient resources^9^ occur in these microbial associations. However, the mechanistic understanding of the dynamics driving community formation remains limited with these approaches, due to the high complexity of natural systems. In addition, the inference of predictive networks in natural bacterial communities faces serious hurdles. For example, spatial heterogeneity of the environment is essentially overlooked, while it is expected to play a significant role in community structure and functioning^10^. An appropriate time scale is also difficult to capture. In particular, whether the community experiences a transient phase or a stationary state at the considered time points is typically unknown. Ultimately, this means that environmental factors are intrinsically impossible to control.

Conversely, experimental models of microbial communities that are highly simplified and limited in size make it possible to manipulate microorganism populations, traits, and the control of environmental parameters^3,11^, as well as to complete experimental replicates. As previously discussed^11,12^, these laboratory microcosms are not intended to reproduce natural communities but rather to uncover general principles operating in field communities as exemplified in the pioneer work of G.F. Gause^13^ who, based on yeast or paramecia co-cultures, described and conceptualized the interspecies interaction mechanisms that led to his theory of competitive exclusion.

Bacterial communities principally live attached to surfaces in nature, forming cell assemblages embedded into an extracellular polymer matrix that ensures their clustering and supports their structure, with major consequences for their development and their different properties^14–18^. These heterogeneous, highly concentrated adherent communities, also called biofilms, are the site of multiple physicochemical gradients^19^. The resulting spatial organization^10,20–24^ is completely lost when samples are harvested from their initial development site for analysis in the laboratory. Recent advances in microscopy imaging^25,26^ and microfluidics^27–29^ have facilitated biofilm in situ explorations and investigations of their spatial features. In addition, there has been an increasing interest in multispecies systems. Experimental approaches dedicated to multispecies biofilms are also beginning to yield reports for a whole range of social interactions and spatial structures in specific models^30–36^. However, due to the difficulty in implementing accurate real-time imaging in composite systems, biofilms are imaged in most studies at given time points using FISH and adaptations of this technique on fixed samples^22,33,37–44^, which can miss or obscure important kinetic information. On the other hand, individual-based or continuum modeling approaches help to formalize mechanisms potentially involved in biofilm structure^45–49^. However, the complexity of adherent community development requires many processes that combine cell biological traits with environmental physicochemical properties that are impossible to fully integrate in the model.

For these reasons, we contend that the kinetic analysis of a multispecies biofilm formation should provide more insight into the mechanistic bases underpinning community establishment and dynamics. We have thus worked to build an experimental model composed of four species of bacteria derived from a natural biofilm that accidentally developed in the industrial context of a milk pasteurization line^50^. Our assembly, which included *Bacillus thuringiensis (Bt)*, *Pseudomonas fluorescens (Pf)*, *Kocuria varians (Kv),* and *Rhodocyclus* sp. *(Rh)*, aimed at a trade-off between simplification, preservation of sufficient diversity, and cultivability. The four species are a priori only related by their ability to coexist spontaneously, which opens up a large spectrum of mechanisms that potentially underlie the formation of the community. Our setup situates the four species in a millifluidic channel under flow^51^, representational of a whole range of living or abiotic environments, such as veins or streams^2^. Made of polydimethylsiloxane (PDMS) and glass, the device provides regulated hydrodynamics and medium supply. In the presence of a growing biofilm, physical and chemical gradients are generated, offering a controlled habitat, complex enough to create environmental diversity. The spatiotemporal development of the composite adherent community is monitored using optical videomicroscopy. We also examined all possible combinations from 1 to 4 of the four species in order to decipher the full community-specific traits.

Here, we demonstrate that the four chosen species form a dynamic adherent community that deterministically reaches its equilibrium after about 30 h of growth. We provide evidence for the arrival of spatial heterogeneity and non-monotonic developmental kinetics, confirming the emergence of complexity in this simplified model based on competitive and physical interspecies interactions. Finally, we discuss the community mode of organization of this experimental model from the perspective of how it can be used to further investigate the response of this system to perturbations.

## Results

### The four species assemble according to a robust temporal sequence and form a community that ultimately reaches dynamic equilibrium

**Fig. 1:**
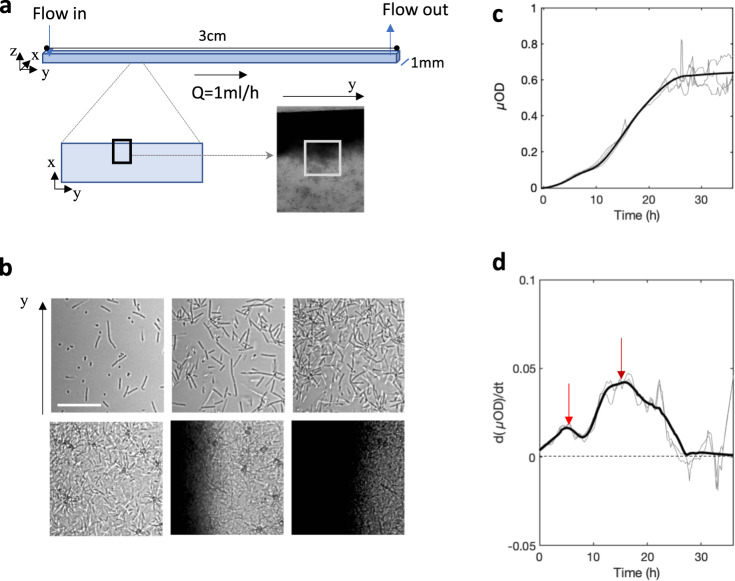
4S biofilm growth under constant flow in the millifluidic channel. **a** A square channel (1 mm × 1 mm and 30 mm in length) continuously fed with growth medium at 1 mL/h was positioned on a microscope stage thermostatically maintained at 30 °C. Images of 0.15 µm^2^ in size, covering nearly half of the width of the channel from the edge to the middle (black rectangle), were taken every 10 min. **b** Zoomed in images corresponding to the gray squares in **a** at time *t* = 10 min (left upper); 2h30 (center upper); 5 h (right upper); 18 h (left lower); 30 h (center lower); and 35 h (right lower), scale bar represents 30 µm (**c**). The µOD—calculated from transmitted light images, i.e., ln(*I*_0_/*I*), and proportional to biomass—as a function of time. The intensity *I* is averaged over all of the pixels in the 0.15 µm^2^ image (black frame in **a**). **d** Derivative of the µOD signal with respect to time. The red arrows show maxima of the expansion rate. Light curves are from three independent biological replicates, and the bold curve is the smoothed average.

*Community formation*: To assess the global development of the attached community under the constant flow of growth medium in the millifluidic channel (Fig. 1a), we acquired time-lapse images (Fig. 1b) and measured the temporal variations of the biomass as reported by the microscopic optical density (Fig. 1c). The kinetics show a biomass increase that levels off after ~25 h. In addition, several inflections were robust across different positions in the same channel, as well as multiple channels and biological replicates. Importantly, these inflections demonstrate the succession of two characteristic temporal phases with peaks sharply delineated by the derivative curve that measures the biofilm expansion rate (Fig. 1d). A first biofilm initiation phase was spread over the first 8 h and included an accelerated growth phase (increasing expansion rate) followed by a damping phase (decreasing expansion rate), with a peak at *t* = 5 h. Next, there was a second expansion phase that exhibited a peak around *t* = 16 h, followed by a kinetic steady-state. Remarkably, the stabilization of the average level of biomass coincided with a marked noise increase in the biomass (Fig. 1c). Movies made from the image stacks clearly show that this noise comes from the frequent passage of biofilm flocs that detach upstream of the observed position (Supplementary Video 1 and Supplementary Video 2). Locally, detachment events can be observed creating gaps that refill in less than an hour (Supplementary Video 3). Thus, the kinetic steady-state signal likely results from the balance between biofilm growth and detachment, consistent with a dynamic equilibrium.

The time-lapse images revealed the evolution in the spatial distribution of the biofilm in the channel (Fig. 1b). Initially, the colonization occurs and progresses uniformly on the bottom surface. Then, in the second expansion phase, there is an accumulation at the channel edges, whereas the central zone remains less densely populated.

#### Steady-state assessment

**Fig. 2:**
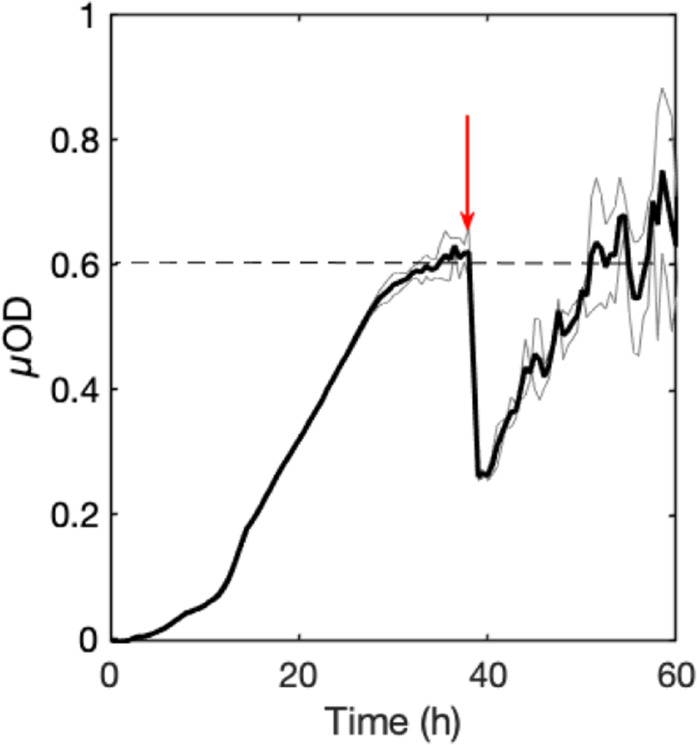
4S biofilm recovery after perturbation. Evolution of the biomass (as reported by µOD) of the 4S biofilm grown in the square channel (1 mm^2^) at 1 mL/h for 38 h at 30 °C, and then partially destroyed by the injection of a 200-µL bubble of air (red arrow). The bold curve shows the average of three distinct positions in the channel (light gray curves).

In order to assess the stability of the community steady-state, we evaluated the ability of the community to recover after a perturbation. For this, we applied a major physical disturbance by injecting a 200-μl air bubble at time *t* = 38 h, which detached approximately half of the attached biomass. Indeed, the μOD was reduced by a factor of 2, returning the biofilm to the level it had reached 20 h earlier, before perturbation (Fig. 2). These results suggest the formation of a stable community^52,53^. However, the high signal-to-noise ratio in the recovery part of the curve, corresponding to the detachment of small flocs, could be indicative of a more physically fragile material that is re-formed after the perturbation.

### A species-specific signal delineates individual kinetics and suggests interspecies coupling

**Fig. 3:**
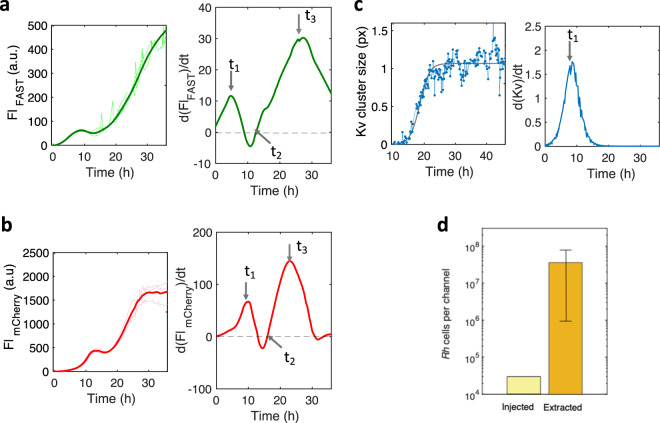
Individual species developments in the 4S adherent community. **a**, **b** Kinetics of the fluorescence intensity, Fl, from *Bt*-FAST (panel A, left side) and *Pf*-mCherry (**b**, left side) in the 4S biofilm with the corresponding Fl curve derivative (**a**, **b**, right side). Fl is the intensity per pixel averaged over the whole image. The arrows on the derivative curves mark the characteristic times *t*_1_, *t*_2_, and *t*_3_ reported in Table 1. **c** *Kv* area detected on transmitted light images as a function of time (left panel, blue line) and corresponding logistic adjustment (gray line) together with its derivative (right panel) (details provided in Supplementary Fig. 9). **a**, **b**, **c** The signals were collected from 0.15 µm^2^ images located as in Fig. 1a (black frame). Light curves are from three independent experiments, and the bold curve is the smoothed average. **d** Bar graph of *Rh* cells injected into the channel (light yellow bar) and recovered after 36 h of growth (dark yellow bar). The same experimental conditions were used as in Fig. 1. Error bar represents standard deviation.

To decipher the features of the 4-species (4S) biofilm development, we investigated the discrete contribution of each species to the community formation. For this, we exploited *Bt* and *Pf* fluorescence signals (Fig. 3a, b) as well as the *Kv* high-contrast signal in transmitted light mode (Fig. 3c), and we counted *Rh* cells at final time points after removing the biofilm from the channel (Fig. 3d). *Bt* and *Pf* fluorescence kinetics exhibited multiphasic curves composed of an initial oscillation followed by an intensive growth phase trending towards a plateau, as already detected on the global transmitted light signal. The derivatization of the kinetics featured the succession of the distinct phases and accentuated the main peaks (Fig. 3a, b), enabling the extraction of three characteristic times for each species: *t*_1_, the maximum of the expansion rate (at the start of damping); *t*_2_, the end of the initial oscillation taken as the point where the derivative becomes positive again; and *t*_3_, the maximum of the recovery, corresponding to the inflexion preceding the plateau (Table 1). Although *Bt* and *Pf* development followed similar kinetics, we observed a shift of several h in the characteristic times related to each species: intriguingly, *Pf* exhibited delays in *t*_1_ and *t*_2_, but with an earlier plateau as compared to *Bt*. In addition, the correspondence of the *Bt* and *Pf* kinetics suggested the existence of a coupling, although the peaks were not fully synchronized.

**Table 1:** Characteristic times of individual species expansion in the 4S biofilm in the whole channel.

Species	t1^a,b^	t2^a,c^	t3^a,d^
*Bt*	4.9 ± 0.2	12.58	27.1 ± 0.3
*Pf*	9.5 ± 0.5	15.9	22.9 ± 0.5
*Kv*	8.5	NA	NA

^a^Times in hours.

^b^Peak time determined at the first maximum of the fluorescence intensity derivative curve.

^c^Time of the expansion rate variation inversion, fluorescence intensity derivative equal to zero.

^d^Peak time at the second maximum.

**Fig. 4:**
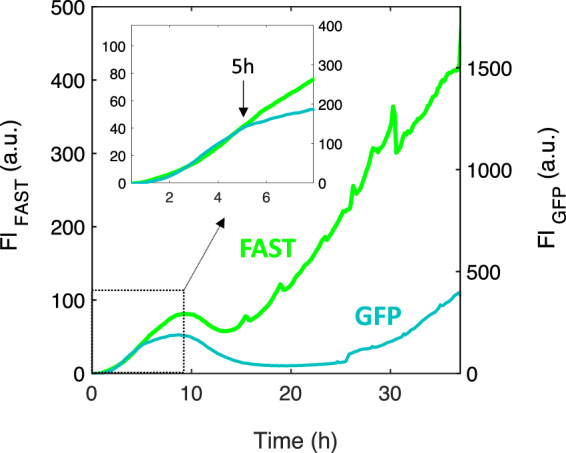
*Bt*-FAST and *Bt*-GFP signals diverge at the characteristic time *t*_1_. Kinetics of the fluorescence intensity, Fl, from *Bt*-FAST and *Bt*-GFP grown in parallel channels in the 4S biofilm. The curves represent the average of at least three independent measurements. The arrow indicates time *t* = 5 h, when the GFP and FAST signals diverged. The insert shows a zoom in on the first 8 h of biofilm formation. The same experimental conditions were used as in Fig. 3a.

In parallel experiments, we monitored the fluorescence signal of *Bt*-GFP in the 4S community and compared it to *Bt*-FAST kinetics in order to obtain an indirect evaluation of O_2_ depletion throughout the biofilm formation. Indeed, as shown in a previous work^54^, the proportionality between the FAST and GFP signals is lost when the environmental O_2_ level decreases below the threshold that enables GFP final maturation and fluorescence. We observed that the *Bt*-GFP fluorescence curve, which is initially superimposable with that of *Bt*-FAST, diverged at time *t* = 5 h (Fig. 4), corresponding to *t*_1_, the first *Bt* growth peak.

At the same time, the *Kv* population developed from initially attached single cells under the form of small clusters, the size of which was taken as a growth index to plot *Kv* expansion kinetics in the community (Fig. 3c). The obtained curve was formally adjusted to a logistic growth curve, with only one characteristic time, *t*_1_, reporting a single growth phase (Table 1). Due to the small cell size, high motility, and absence of labeling, the temporal evolution of the *Rh* population could not be accurately determined. The contribution of *Rh* was therefore evaluated by enumerating the cells after biofilm extraction at the final stage, revealing that the *Rh* population successfully thrived in the community, as indicated by its multiplication by three logs during the biofilm development (Fig. 3d). In comparison, *Bt* and *Pf* multiplied by ~3 and 4 logs, respectively (Supplementary Fig. 1).

These results suggest the existence of a coupling between the *Bt* and *Pf* populations, in which they both exhibit two successive growth phases, similar to the global biomass kinetics. By contrast, the *Kv* population only exhibits a one-phase expansion. The individual kinetics show that the four species were present all throughout the biofilm development. Oxygen depletion was deduced in correlation with the first peak of the community.

### The 4S community displays species-specific spatial distribution

To document spatial heterogeneity emergence in the community, we examined the evolution of the species spatial distribution throughout the community expansion.

**Fig. 5:**
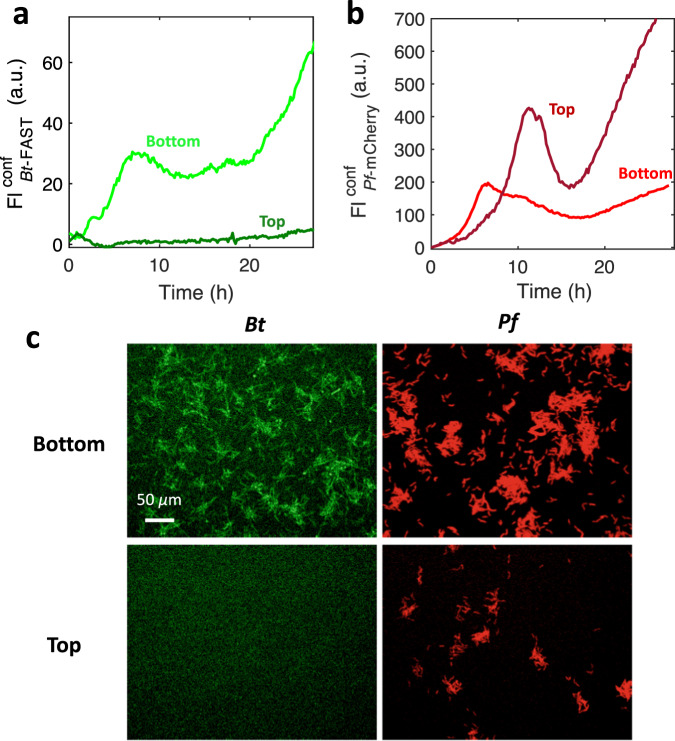
Surface-dwelling characteristics of *Bt* and *Pf* in the channel: bottom vs. top surface colonization. Confocal fluorescence intensities of **a** *Bt* and **b** *Pf* recorded with a focus on the bottom surface (light green (**a**) and light red (**b**)) and on the top surface (dark green (**a**) and dark red (**b**)). Axial resolutions are 5 and 5.8 µm for FAST and mCherry signals, respectively. **c** Confocal images recorded at time *t* = 3 h on the bottom surface (upper panels) and on the top surface (lower panels) for *Bt* (left panels) and *Pf* (right panels). The same experimental conditions were used as in Fig. 1.

First, we investigated the species vertical distribution. For this, a series of confocal microscopy acquisitions were performed to selectively image the bottom and top surfaces of the channel, whereas epifluorescence recordings were made to capture the signal from the whole channel height. We found that *Bt* strictly dwelled on the bottom surface of the channel, whereas *Pf* colonized both the bottom and top surfaces (Fig. 5). The *Pf* bottom and top surface populations displayed similar kinetic profiles, although the first oscillation was delayed by 5 h on the top surface in comparison to the bottom surface. These acquisitions also showed that *Bt* and *Pf* exhibited synchronous kinetics on the bottom surface (see Tables 1 and 2). Moreover, transmitted light observations showed that *Kv* locations were also limited to the bottom surface (Supplementary Fig. 2). Thus, *Pf* appears to share the bottom surface with the other species, but also colonizes a specific niche on the top surface.

**Table 2:** Pf bottom vs. top surface kinetics.

Localization	t1^a^	t2^a^	t3^a^
Pf bottom	5.04	15.92	23.08
Pf Top	11.09	16	NA

^a^Times defined as in Table 1.

**Fig. 6:**
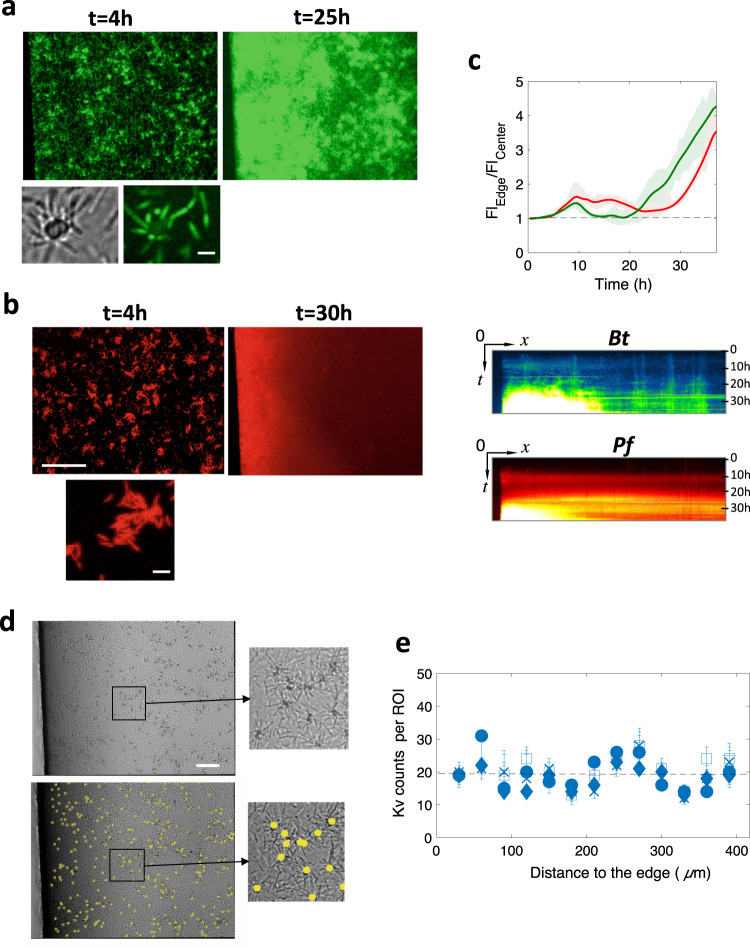
Spatial transition to the channel edge. Fluorescence images of *Bt* (**a**) and *Pf* (**b**) populations in the 4S biofilm, taken in the initial colonization phase (left image) and after the spatial transition (right image); scale bar represents 100 µm. The small bottom images are zoomed-in details showing local heterogeneities of the globally uniform distribution in the initial colonization phase; scale bars represent 5 µm. Graph of the ratio as a function of the fluorescence intensity time at the channel edge (Fl_edge_) to that at the channel center averaged on ROIs (20 µm wide) located at 20 and 360 µm from the channel wall, respectively; *Bt* in green and *Pf* in red (**c**, upper panel). Kymographs of *Bt* (green blue) and *Pf* (Red) spatial distribution are presented (**c**, lower panel). **d** Detection of *Kv* cells and clusters in the 4S biofilm using the NIS dark-spot detection tool (details provided in the Supplementary Note 3). Images correspond to time *t* = 14 h. In the bottom panel, the detection results are overlaid on the original image shown in the upper panel; scale bar is 50 μm. **e** *Kv* spots were then counted in 30-μm wide ROIs from the edge to the center of the channel for time *t* = 10 min (⎕); 20 min (●); 7h30 (×); 6h30 (▢); and 32 h (+). Error bars report the error of 10% made on spot counts as evaluated by comparing automatic and visual detection.

Next, based on the spatial distribution shift of the global biomass we reported above (Fig. 1b), we decided to examine the lateral spatial distribution of the individual species. During the first 5 h of colonization, both *Bt* and *Pf* developed a uniform distribution along the x and y axes on the bottom surface, before initiating a progressive shift towards the edges of the channel (Fig. 6a, b). In order to quantify this shift, we defined two 20 µm-wide regions of interest (ROIs), ROI_Edge_ and ROI_Center_, located at 20 and 360 µm from the channel wall, respectively. Then, we calculated the ratio of their mean fluorescence intensities, FL_Edge_/FL_Center_, which provides the intensity of the spatial shift. The evolution of this ratio during the biofilm formation along with representative kymographs are shown in Fig. 6c. These data indicate an even distribution within the first 5 h, followed by a trend to preferentially populate the edge of the channel emerging between 5 and 10 h, and finally a clear-cut spatial distribution transition towards the channel edge arising shortly after the onset of the second growth phase.

The *Kv* spatial distribution was analyzed from images of the (x, y) coordinates of the *Kv* cells and clusters attached on the channel bottom surface (Fig. 6d, e). We found that the discrete *Kv* locations were evenly distributed over the bottom surface all throughout the biofilm development, despite short-length scale heterogeneities. This indicates that *Kv* did not participate in the spatial transition supported by *Bt* and *Pf*, consistent with its kinetic profile, which is also clearly distinct from that of *Bt* and *Pf*. The synchronized *Bt* and *Pf* spatial transition reinforces the hypothesis of the coupling of these two populations in the community on the bottom surface. In addition, the temporal correspondence of this spatial transition with the second growth phase indicates that this apparent translocation corresponds to a geographically restricted growth. All species coexisted on the bottom surface, whereas *Pf* was also able to accommodate a private niche on the top surface.

### Local dynamics analysis reveals both static and highly mobile species

To better characterize the initiation phase of the biofilm, we examined the local dynamics of the four species within the first 6–7 h of the biofilm growth. During this time period, corresponding to the first growth phase, the colonized fraction of the surface is small enough to allow delineation of individual objects.

**Fig. 7:**
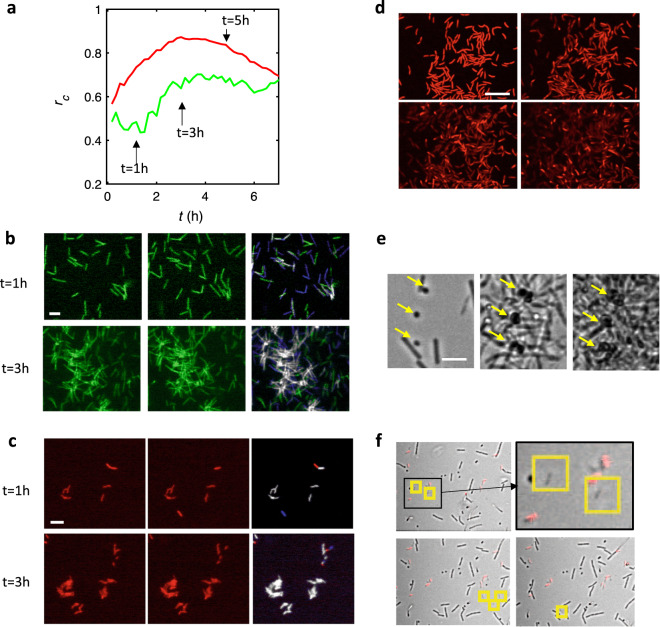
Species exhibit distinct local dynamics in the 4S community. **a** The consecutive frames correlation coefficient calculated from time-lapse recordings of *Bt*-FAST (green curve) and *Pf-*mCherry (red curve) in the 4S biofilm community. Colocalization maps of *Bt* (**b**) and *Pf* (**c**) cells reveal spatial self-overlap between time *t* and *t*′ = *t* + 10 min, taken at *t* = 1 h (upper panels) and *t* = 3 h (lower panels). Light gray pixels correspond to non-moving cells (pixels unchanged between *t* and *t*′), while blue pixels correspond to newly appeared cells (pixels that were dark at *t* and subsequently light at *t*′), and magenta pixels correspond to removed cells (pixels that were light at *t* and subsequently dark at *t*′); scale bar represents 10 µm. **d** Confocal images of *Pf*-mCherry in the 4S community taken at *t* = 6h40 (left upper); 7 h (right upper); 7h30 (left lower); and 8 h (right lower), showing full detachment of the cell groups; see also colocalization maps in Supplementary Fig. 3; scale bar represents 5 µm. **e** Transmitted light images of the 4S biofilm taken at time *t* = 1 h (left); 12 h (center); and 37 h (right), showing fixed localizations of *Kv* (indicated by the yellow arrows) all throughout the biofilm growth; scale bar represents 5 µm. **f** Successive frames (left upper; left lower, right lower) of the 4S biofilm in transmitted light and red fluorescence taken from *t* = 1 h (left upper) at 10-min intervals. The upper right frame is a zoom in of left upper quadrant. Yellow squares indicate the *Rh* cells, illustrating their high dynamics with different positions on each frame; scale bar represents 10 µm.

The dynamics of the fluorescent species *Bt* and *Pf* were extracted from stacks of fluorescent images to calculate the correlation coefficient, *r*_*c*_, between consecutive frames (Fig. 7a). *Bt* colonization exhibited a correlation coefficient of *r*_*c*_ <0.5 (Fig. 7a), demonstrating a loose attachment on the surface that was corroborated by the colocalization maps of successive images (Fig. 7b). After about 2 h, the correlation coefficient increased, coinciding with the formation of fluorescent asters resulting from the progressive aggregation of *Bt* around *Kv* cells, which physically stabilized the connected *Bt* cells while other cells remained highly mobile (Supplementary Video 4).

*Pf* cells exhibited a strong surface anchoring characterized by correlation coefficients of 0.8–0.9 after 2 h of colonization (Fig. 7a). The colocalization maps show that most of the initial attachments generated microcolonies (Fig. 7c). At time *t* ≈ 5 h, corresponding to the first peak of development in the *Pf* population on the bottom surface, *r*_*c*_ decreased while *Pf* cells simultaneously started to detach, shifting from firmly attached to essentially detached cells in less than 2 h (Fig. 7d and Supplementary Fig. 3). Notably, a similar detachment occurred on the top surface, although it was consistently delayed by about 6 h in comparison to the shift in peaks described above.

*Kv* dynamics were assessed from the localization maps used to evaluate its spatial distribution. The stable attachment of isolated cells randomly dispersed over the whole bottom surface of the channel was observed within minutes of biofilm initiation. As the biofilm grew, compact clusters of several cells formed essentially at the same location as the initial attachment (Fig. 7e). Indeed, at least 90% of the *Kv* clusters originated from these single-cell initial anchors. A correlation coefficient of 0.94 was calculated from the position map (Supplementary Fig. 4).

*Rh* was difficult to monitor due to its high dynamics. Several cells could be visually detected within the first images, although their surface-dwelling time was below the 10 min of the frame period, meaning that *Rh* cells were never found at the same location in consecutive frames (Fig. 7f). Therefore, the correlation coefficient for *Rh* within the colonization period was assigned as ‘0’.

The colonization dynamics of the four species ranged from highly dynamic for *Rh* (*r*_*c*_ = 0) to completely fixed for *Kv* (*r*_*c*_ = 0.95). The *r*_*c*_ values, which measure the bacterial residence on the surface in the presence of the flow, reflect the significantly distinct capacities of the four species to adhere to the surface. The correlation profile of *Bt*, whose *r*_*c*_ value increased upon association with *Kv*, also reveals how physical interspecies interaction can improve the colonizing potential of poorly adhesive species.

### 4S combinatorics highlights community-specific traits

Next, we asked what are the community-specific traits of the 4S biofilm. For this, we designed a combinatorial approach in which all possible sub-combinations of the four species were injected into different parallel channels (14 in total). Then, we collected the optical signals of all of the channels during the first 36 h following channel seeding. The recordings of the different mixes are exhaustively reported in the Supplementary Information (Supplementary Fig. 5), while we describe below the most significant features for the interpretation of the community traits.

#### Bt improves its colonization and physical stability in the 4S biofilm

**Fig. 8:**
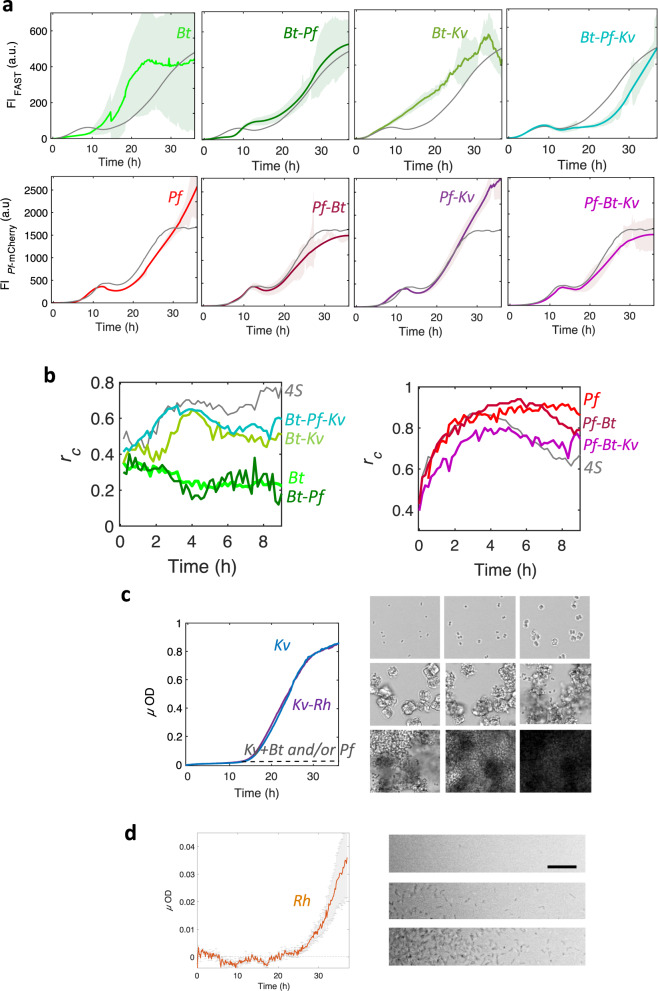
Species combinatorics highlights community-specific traits. **a** Fluorescence signals of *Bt* (upper row) and *Pf* (lower row) in various sub-combinations of the four species: single-species (left column), pairs (second and thrird column), and triplets (right colum). **b** The consecutive frames correlation coefficient for *Bt* (left) and *Pf* (right) for the different combinations are provided on the figure. The experimental conditions other than the biofilm composition were the same as in Fig. 3. **c** Micro-optical density of the *Kv* single-species biofilm (in blue) and the *Kv-Rh* pair (in purple; graph on the left); the dashed line shows the level of *Kv* development in the presence of *Bt* alone, *Pf* alone, and *Pf* and *Bt* together, deduced from the *Kv* area determination and the mean µOD of a *Kv* cluster. Images on the right are snapshots of the single-species biofilm taken at time *t* = 10 min (left upper); 3h30 (center upper); 9 h (right upper); 15 h (left center); 16h30 (center center); 18h30 (right center); 23h30 (left lower); 27h30 (center lower); and 30 h (right lower). **d** Micro-optical density of *Rh* single-species biofilm (graph on the left) and corresponding snapshots taken at time *t* = 10 h (upper); 25 h (center); and 37 h (lower). The curve of the studied combination in each panel (bold color line) is the average of at least three independent samples and appears shaded with the standard deviation of the data set. The curve of the corresponding species in the 4S community is plotted in gray for comparison. Scale bars represent 20 µm.

The biofilm built by *Bt* alone exhibited several characteristics distinct from the 4S biofilm. In particular, the initial population oscillation was absent, replaced by a 10–12-h-long lag phase where the cell amount at the surface remained low (Fig. 8a). We also observed that local dynamics were characterized by correlation coefficient values below 0.4 (Fig. 8b), indicating that *Bt* cells essentially did not attach to the surface in the absence of the other species. After 10 h, the fluorescence signal significantly increased, revealing a colonization phase similar to the second phase displayed by *Bt* in the 4S biofilm, but affected by a strong instability. The standard deviation was on the order of the signal itself, due to massive random detachment events resulting in important variations in the signal (Supplementary Video 5). In addition, the single-species *Bt*-FAST and *Bt*-GFP biofilms exhibited fully superimposable fluorescence signals, indicating that no O_2_ depletion occurred in this situation (58) (Supplementary Fig. 6). In the presence of *Pf*, the *Bt* development profiles did not drastically change compared to that of *Bt* alone, except that the colonization phase appeared to be delayed for several hours compared to the expansion of *Bt* alone (Fig. 8a). The standard deviation of the data sets was also three times less than for *Bt* alone, suggesting that a more physically stable biofilm had formed. The biofilm built by the *Bt-Kv* pair (Fig. 8a) confirmed the specific *Bt*-*Kv* physical interaction leading to the formation of *Bt* asters around *Kv* (Fig. 6a and Supplementary Video 4), as well as the physical grip effect that promoted *Bt* early colonization, as revealed by an increase in the fluorescence intensity and the consecutive frames correlation coefficient (Fig. 8b). In the 3-species *Bt-Pf*-*Kv* community, the *Bt* development profile sequentially combined (1) the traits of the *Bt-Kv* pair, in which the initial phase promoted *Bt* growth, and (2) the traits of the *Bt-Pf* pair, in which the second phase induced latency (Fig. 8a). This 3-species community closely resembles the 4S community, with its initial oscillation and second growth phase. However, in contrast to the *Bt-Pf* and 4S communities, the 3-species *Bt-Pf*-*Kv* community exhibited an extended *Bt* second phase latency.

#### Pf undergoes a Bt-imposed dynamic equilibrium

The various combinations of *Pf* exhibited the same main traits as revealed in the 4S biofilm, except for an increase in the dynamic equilibrium that was absent from any combination lacking *Bt* (Fig. 8a). Indeed, *Pf* appears to be the least impacted species in the various combinations, as it is simply forced to equilibrium by the presence of *Bt* after 30 h of combined growth.

#### Kv development is inhibited in the 4S community

When injected alone into the channel, the *Kv* population formed a profuse single-species biofilm. The cells immediately adhered to the bottom of the glass slide and multiplied in situ, forming compact cubic clusters during the first 10 h of biofilm formation, as described above for the 4S community (Fig. 8c). Whereas the *Kv* clusters were halted in this stage in the 4S community, they continued growing exponentially in the single-species mode, forming larger cohesive clusters (composed of around a hundred cells) before bursting again into single cells that were partially removed by the flow, and partially redirected to the surface, where they efficiently formed a thick biofilm (Fig. 8c, Supplementary Video 6). We found that the *Kv* population was inhibited by the presence of *Bt* alone, *Pf* alone, and both *Bt* and *Pf* (Fig. 8c and Supplementary Fig. 7), just as they were in the 4S community (Supplementary Fig. 2). Notably, *Kv* cells extracted from two-species and three-species biofilms with *Bt* and/or *Pf* were recovered by plating on agar and forming colonies, indicating that they were still viable.

#### Rh, the community-neutral element

*Rh* alone exhibited poor colonization abilities, and a thin layer of attached cells concentrated at the edge of the channel emerged after about 25 h (Fig. 8d). The contribution of *Rh* in mixed communities was always optically overwhelmed by the other species, and could not be characterized in real-time. Although their presence was confirmed by channel extraction counting, *Rh* cells did not alter any characteristics of the other species when included in multispecies communities (Supplementary Fig. 5).

## Discussion

We report here a real-time analysis of a four-species bacterial assembly, composed of a more complex natural community (50) and grown under constant flow of growth medium in a laboratory millifluidic device. The simple and defined structure of our setup provided a controlled environment, the heterogeneity of which was sufficient to allow behavioral complexity to emerge.

We showed that the 4S assembly forms a surface-attached community displaying a robust dynamic equilibrium, with a biomass reconstituted in about 36 h after physical perturbation. The 4S community developed according to a deterministic mechanism involving timed spatiotemporal phases of expansion and recession. However, two species, *Pf* and *Bt*, clearly dominated the developmental program. Remarkably, while the four species coexisted on the bottom surface, a specialist niche emerged on the top surface, exclusively accommodating *Pf*, which was not constrained by gravity or bottom surface assignation. Considering the channel dimensions (a 1 mm^2^ cross-section) and the flow rate (1 ml/h), characteristic times of 2 min for advection and 10 min to several hours for molecular diffusion in the height of the channel can be evaluated from the Stokes–Einstein equation^55^. Therefore, the bottom and top surface habitats can be regarded as independent as well as their respective biomes, reminiscent (on a much smaller scale and range) of the biogeographical sorting that occurs in nature^56^.

On the bottom surface, the *Pf* and *Bt* populations appeared to be closely correlated in both time and space. Our results support two distinct mechanisms of coupling. The first one, which governs the first 25 h of biofilm formation, is mediated by the environment and interpreted as a coupled response to the depletion in O_2_ of the environment, principally resulting from *Pf* population growth. This hypothesis is supported by the comparison of *Bt*-GFP and *Bt*-FAST fluorescence signals in the 4S and single-species communities. Consistently, *Pf* development kinetics exhibited very similar profiles in the 4S and single-species biofilms within the first 25 h of formation, suggesting a predominant imprint of *Pf* on the environment. In this scenario, the O_2_ depletion of the environment is assumed to drive *Pf* detachment and its spatial transition to the channel edges. Indeed, we know from previous experiments that renewal of O_2_ supplies occurs principally at the channel edges in our device due to the permeability of PDMS to O_2_^51,54^. This results in the selective growth of aerobes in regions of higher O_2_, where they compete for the same resource. Here, we highlight a mechanism in which the spatial distribution is governed by the shaping of the environment by one of the species. Such a resource-driven spatial organization might be a key principle in the emergence of spatial structure in complex biofilms. This has also been previously described in mixed genotype colonies of *Pseudomonas aeruginosa*^57^. Nevertheless, the reciprocal influence of spatial distribution on social interactions has also been described^40^, confirming that no unequivocal link between social behavior and spatial structure can be established^21^. Moreover, we provide evidence that spatial structure evolves throughout biofilm development.

The second dependency is revealed by the expression of a *Bt–Pf* interaction is also found among the characteristics of the dynamic equilibrium established after 30 h of 4S biofilm growth. Based on the combinatorial assembly of multispecies communities, it is clear that *Bt* forces *Pf* to a plateau resulting from balanced growth and detachment, in contrast to the continuous expansion that *Pf* exhibits in its pure biofilm. The presence of *Pf* also provides better physical stability to the co-existing population of *Bt*, as shown by the significant decrease in the standard deviation of the *Bt* signal in the presence of *Pf*, and the reduction in the number of peaks due to detachment events.

These changes in the stability of the biofilm raise questions about the assembly of the extracellular matrix in a multispecies context, a topic that is only starting to be investigated^58^. Indeed, biofilm stability is closely related to extracellular matrix properties, such as the degree of cross-linking^59^. The larger spectrum of components brought together in a multispecies environment might offer novel opportunities to form cross-links. According to this hypothesis, assembly of the matrix is a community activity that occurs extracellularly, as reported previously for *Candida albicans* biofilms^60^. This illustrates how spatial reorganization can lead to the emergence of novel properties, reinforcing the view of a critical role for spatial distribution on biofilm properties^20,22,24,61,62^.

*Bt* and *Kv* displayed a specific physical interaction that enabled several *Bt* cells to attach to a *Kv* cluster and benefit from the strong affinity of *Kv* cells for the bottom surface of the channel, in particular during the initial phase of colonization when *Bt* alone barely forms any transient ties with the surface. This interspecies physical attachment has been largely overlooked in the analyses of multispecies community structures, in contrast to social interactions mediated by diffusible factors, such as quorum sensing or metabolite tradeoffs^46,63–65^, with the exception of oral biofilms^66^. A better understanding of the impact that these specific bindings have on biological functions, as well as additional information about their diversity and their evolutionary profile, could provide new perspectives to help understand multispecies biofilms.

The *Kv* population experienced a major limitation of its development in mixed communities containing *Bt* alone, *Pf* alone, and both *Bt* and *Pf*. Since both *Bt* and *Pf* inhibit *Kv* biofilm development without killing it, it is tempting to postulate the existence of an environmental effect driven by the development of neighboring species, rather than a specific interspecies interaction. However, at this stage, there are no clues as to which parameter(s) could be involved. In particular, the inhibition did not reveal any positional dependence, ruling out a link to O_2_ supply, as assumed in the explanation of *Bt* and *Pf* behaviors. In addition, a steric hindrance effect, due to the faster growth of *Bt* and *Pf*, cannot be excluded.

Finally, *Rh* appears to be the independent partner of the community. When grown as a single-species biofilm, this highly motile bacteria were found to develop a poor attachment. Nevertheless, it still multiplied and persisted in the 4S community in an apparently fully neutral mode, having no detectable interaction with any other species. It must be noted that we did not obtain strong data for *Rh*, since it could not be tracked in the multispecies communities. However, we decided to conserve it in the consortium as an example of coexistence without interactions, anticipating a possible role for this neutral element in disturbed environmental conditions.

We conclude that our composite biofilm reaches its dynamic equilibrium based on local cell concentration, environmental physicochemical properties that are constantly reshaped by the community development itself, and a species fitness altered by surface attachment. From a conceptual perspective, this community could be considered as individualistic in the sense of Gleason^67^, but also as a limiting case of an organismic continuum, as theoretically proposed by Liautaud et al.^68^. Indeed, species individually seek out to optimize their own growth but doing this, they shape their common environment, which provides a certain level of collective behavior.

This multispecies biofilm emerges as the result of chance (a given species in a given environment) and necessity (individual species adaptation) deterministically leading to a unique community. Importantly, the emergent properties could not be predicted from individual traits, instead involving a form of biofilm sociobiology that does not require cells to communicate with one another using specialized signaling molecules^69^.

The operational experimental model and assembly principles, we present here, should be useful for investigating a multispecies bacterial community response to perturbations, or even for developing new strategies that can manipulate biofilm functions in several fields including health, agriculture, and the environment.

## Materials and methods

### Bacterial strains and culture conditions

*Bt* is a 407 Cry^−^ strain^70^. The fluorescent variants *Bt*-FAST and *Bt*-GFP were genetically engineered to express either the protein FAST^71^ or GFP under the control of the constitutive promoter P*sar*. *Pf* is an mCherr*y*-expressing strain (WCS365 containing pMP7605)^72^, gifted by E.L. Lagendijk from Leiden University (The Netherlands). *Kv* (CCL56) and *Rh* (CCL5) were isolated from a biofilm formed on a gasket in a milk pasteurization line^50^. The strains were routinely cultivated at 30 °C on M1 medium (Supplementary Table 1).

### Millifluidic device

We microfabricated millifluidic channels 30 mm in length, 1 mm in width, and 1 mm in height. A PDMS mixture (RTV615A+B; Momentive Performance Materials) was poured at ambient temperature in a polyvinyl chloride home-micromachined mold and left to cure at least 3 h in an oven set at 65 °C. Then, the recovered templates were drilled for further plugging of adapted connectors and tubings. PDMS templates and glass coverslips were then cleaned using an oxygen plasma cleaner (Harrick) and immediately bound together to seal the channels. For connections, we used stainless steel connectors (0.013″ ID and 0.025″ OD) and microbore Tygon tubing (0.020″ ID and 0.06″ OD) supplied by Phymep (France). The thin metallic connectors provide a bottleneck in the flow circuit, which prevents upstream colonization. The medium was pushed into the channels at a controlled rate using syringe pumps for the 36–40 h of the experiment. Up to 12 channels can be run and monitored in parallel. The whole experiment was thermostatically maintained at 30 °C.

### Biofilm formation

#### Initiation

Approximately 1.2 × 10^5^ cells from exponentially growing cultures of each species were mixed and immediately injected directly into the PDMS channels using a syringe equipped with a 22 G needle before connecting the tubings. Next, the cells were allowed to settle for 1h30 before starting the medium flow. Nutrient flow triggering was initiated at time *t* = 0. For biofilm growth, we used MB medium, an adaptation of M1 medium (Supplementary Table 1). Overnight cultures in M1—seeded with a single colony from M1-agar plates—were grown at 30 °C under agitation. Exponential phases were obtained from dilutions in M1of these overnight cultures incubated at 30 °C under agitation up to defined ODs depending on the cultured species (Supplementary Table 2).

#### Attached community development

The flow rate of 1 mL/h imposed a mean advection characteristic time $\tau _a$ of 2 min, while in the Stokes–Einstein approximation the characteristic sedimentation time $\tau _s$ was on the order of 1 h, and the diffusion characteristic time $\tau _d$ was on the order of 1 h for motile bacteria to several hours for non-motile cells. This implies that, on average, bacteria suspended in the channel will be continuously and rapidly washed out by the flow, essentially leaving attached cells that reside and divide in the channel together with a minority of freshly detached cells. Microscope time-lapse imaging of the channel bottom surface was initiated several minutes before triggering the flow at time *t* = 0.

### Microscope imaging

#### Microscope

We used an inverted NIKON TE300 microscope equipped with motorized x, y, z displacements and shutters. Images were collected using a 20 × S plan Fluor objective (NA 0.45 WD 8.2–6.9 mm). Bright field images were collected in direct illumination (no phase). Fluorescence acquisitions were performed using either the green channel filters for GFP and FAST:HBR-2,5-DM (Ex. 482/35, DM 506 Em. FF01-536/40), or the red filter for m-Cherry (Ex 562/40 nm DM 593 Em. 641/75). Excitation was performed using an LED box (CoolLed pE-4000).

#### Epifluorescence

We collected fluorescence signals by focusing the image on the bottom surface. Due to the small numerical aperture (0.45) of the objective (20x) and the 1-mm channel height, we collected the signal from the whole channel height (Supplementary Fig. 8).

#### Confocal microscopy

In certain cases, we also collected confocal images using a spinning disk Crest X light V2 module (Gataca, France distribution) with an axial resolution of 5.8 µm.

### Image acquisition

We used a Hamamatsu ORCA-R2 EMCCD camera for time-lapse acquisitions of 1344 × 1024 pixel images with 12-bit gray level depth (4096 gray levels), and captured an *xy* field of view of 330 µm × 430 µm. Bright field and fluorescence images were typically collected for 36 h at a frequency of six frames per hour. Excitation LEDs were set at a 50% power level, and exposure times were 50 or 500 ms for the green emissions (for GFP and FAST, respectively) and 800 ms for the red emissions.

### Image analysis

#### Image intensities

Time-lapse images were analyzed to derive the kinetics of biomass accumulation in the channel based on microscopic optical density (µOD) measured from transmitted light images according to µOD = ln (*I*_*0*_/*I*), where *I*_*0*_ is the intensity recorded on a channel filled with medium only and *I* is the intensity recorded on a channel containing a growing biofilm^51^. Image intensity per pixel averaged for the whole image or on defined ROIs was collected using the NIKON proprietary software NIS. Subsequently, the data sheets edited by NIS were exported to MATLAB for further analysis of biofilm development kinetics and determination of growth parameters. *Bt* and *Pf* expansion kinetics were measured from time-lapse fluorescence intensity images in their respective optical channels. Background was subtracted using the contribution to the fluorescence intensity of a channel of medium in the absence of bacteria. All curves were averaged over at least three independent replicates.

#### Kv delineation

*Kv* cells and clusters in the community were delineated using transmitted light image thresholding and morphological descriptor filtering with the NIS smart thresholding tool. A cell-contrast-based detection using a combination of automatic and user visual approaches was devised to cope with the evolution of the image texture as biofilm grows. To take into account the shading of the image edge produced by the channel side wall which decreased the level of the background, we used two sets of binarization parameters (Supplementary Fig. 9a, b) and a circularity filter (circularity ($(4\pi a/p^2) > 0.58$), where *a* is the object area and *p* its perimeter. The (x, y) coordinates of all the detected objects are stored with their binarization parameters and sorted in MATLAB. The accuracy of the detection is then evaluated by comparing for characteristic data sets the results of the automatic detection with a user visual detection which provided a cumbersome but accurate counting. We found differences comprised between ±2% for the shortest times where the detection is facilitated by the cells dispersion up to 10% as the crowding increased (Supplementary Fig. 9b). Above 10 h, we implemented a visually-assisted semi-automatic detection strategy consisting in choosing more restrictive binarization parameters which missed about 30% of the *Kv* clusters completed with a user-based visual detection correcting the errors (Supplementary Fig. 9c, d). On the longer time scale, the spotting of the *Kv* cells at the edge of the channel was hindered by the biofilm growth and no accurate data could be collect at small x (<100 µm) and longer times (>20 h).

#### Pearson’s correlation coefficient

Fluorescent species dynamics were evaluated by calculating *r*_*c*_, the Pearson’s correlation coefficient, from consecutive frames of biofilm growth movies using ImageJ 1.52k correlator^73^. Perfectly identical images have a coefficient of 1, whereas completely distinct images are close to 0 (Supplementary Fig. 10).

### Reporting summary

Further information on research design is available in the Nature Research Reporting Summary linked to this article.

## Supplementary information

Supplementary Video 1

Supplementary Video 2

Supplementary Video 3

Supplementary Video 4

Supplementary Video 5

Supplementary Video 6

Supplementary Information

Reporting Summary

## Data Availability

The data that support the findings of this study are available from the corresponding author upon reasonable request.

## References

[1] Falkowski PG, Fenchel T, Delong EF (2008). The microbial engines that drive Earth’s biogeochemical cycles. Science.

[2] Battin TJ, Besemer K, Bengtsson MM, Romani AM, Packmann AI (2016). The ecology and biogeochemistry of stream biofilms. Nat. Rev. Microbiol..

[3] Benton TG, Solan M, Travis JM, Sait SM (2007). Microcosm experiments can inform global ecological problems. Trends Ecol. Evol..

[4] Faust K, Raes J (2012). Microbial interactions: from networks to models. Nat. Rev. Microbiol..

[5] Ponomarova O, Patil KR (2015). Metabolic interactions in microbial communities: untangling the Gordian knot. Curr. Opin. Microbiol..

[6] Strom SL (2008). Microbial ecology of ocean biogeochemistry: a community perspective. Science.

[7] Morris BEL, Henneberger R, Huber H, Moissl-Eichinger C (2013). Microbial syntrophy: interaction for the common good. FEMS Microbiol. Rev..

[8] Moscoviz R, Flayac C, Desmond-Le Quemener E, Trably E, Bernet N (2017). Revealing extracellular electron transfer mediated parasitism: energetic considerations. Sci. Rep..

[9] Watkins ER, Maiden MC, Gupta S (2016). Metabolic competition as a driver of bacterial. Future Microbiol.

[10] Hart SP, Usinowicz J, Levine JM (2017). The spatial scales of species coexistence. Nat. Ecol. Evol..

[11] Jessup CM (2004). Big questions, small worlds: microbial model systems in ecology. Trends Ecol. Evol..

[12] Konopka A, Lindemann S, Fredrickson J (2015). Dynamics in microbial communities: unraveling mechanisms to identify principles. ISME J..

[13] Gause G (1932). Experimental studies on the struggle for existence: I. Mixed population of two species of yeast. J. Exp. Biol..

[14] Geesey GG (2001). Bacterial behavior at surfaces. Curr. Opin. Microbiol..

[15] Flemming HC (2016). Biofilms: an emergent form of bacterial life. Nat. Rev. Microbiol..

[16] Hall-Stoodley L, Costerton JW, Stoodley P (2004). Bacterial biofilms: from the natural environment to infectious diseases. Nat. Rev. Microbiol..

[17] Costerton JW (1995). Overview of microbial biofilms. J. Ind. Microbiol..

[18] van Gestel J, Kolter R (2019). When we stop thinking about microbes as cells. J. Mol. Biol..

[19] Stewart PS, Franklin MJ (2008). Physiological heterogeneity in biofilms. Nat. Rev. Microbiol..

[20] Kim HJ, Boedicker JQ, Choi JW, Ismagilov RF (2008). Defined spatial structure stabilizes a synthetic multispecies bacterial community. Proc. Natl Acad. Sci. USA.

[21] Nadell CD, Drescher K, Foster KR (2016). Spatial structure, cooperation and competition in biofilms. Nat. Rev. Microbiol..

[22] Bridier A (2017). Spatial organization plasticity as an adaptive driver of surface microbial communities. Front. Microbiol..

[23] Cutler, N. A., Chaput, D. L., Oliver, A. E. & Viles, H. A. The spatial organization and microbial community structure of an epilithic biofilm. *FEMS Microbiol. Ecol.* **91**, fiu027 (2015). 10.1093/femsec/fiu027 25764559

[24] France MT, Forney LJ (2019). The relationship between spatial structure and the maintenance of diversity in microbial populations. Am. Nat..

[25] Azeredo J (2017). Critical review on biofilm methods. Crit. Rev. Microbiol..

[26] Neu TR (2010). Advanced imaging techniques for assessment of structure, composition and function in biofilm systems. FEMS Microbiol Ecol..

[27] Wessel AK, Hmelo L, Parsek MR, Whiteley M (2013). Going local: technologies for exploring bacterial microenvironments. Nat. Rev. Microbiol..

[28] Rusconi R, Garren M, Stocker R (2014). Microfluidics expanding the frontiers of microbial ecology. Annu Rev. Biophys..

[29] Burmeister, A. et al. A microfluidic co-cultivation platform to investigate microbial interactions at defined microenvironments. *Lab Chip* **19**, 98–110 (2019) 10.1039/c8lc00977e 30488920

[30] Moons P, Michiels CW, Aertsen A (2009). Bacterial interactions in biofilms. Crit. Rev. Microbiol..

[31] Niu, B. & Kolter, R. Quantification of the Composition dynamics of a maize root-associated simplified bacterial community and evaluation of its biological control effect. Bio Protoc. 8, 2885 (2018). 10.21769/BioProtoc.2885 PMC6095191 30123815

[32] Alnahhas RN (2019). Spatiotemporal dynamics of synthetic microbial consortia in microfluidic devices. ACS Synth. Biol..

[33] Liu W (2016). Interspecific bacterial interactions are reflected in multispecies biofilm spatial organization. Front. Microbiol..

[34] Momeni B, Brileya KA, Fields MW, Shou W (2013). Strong inter-population cooperation leads to partner intermixing in microbial communities. Elife.

[35] Ratzke C, Gore J (2016). Self-organized patchiness facilitates survival in a cooperatively growing Bacillus subtilis population. Nat. Microbiol..

[36] Ren D, Madsen JS, Sorensen SJ, Burmolle M (2015). High prevalence of biofilm synergy among bacterial soil isolates in cocultures indicates bacterial interspecific cooperation. ISME J..

[37] Almeida C, Azevedo NF, Santos S, Keevil CW, Vieira MJ (2011). Discriminating multi-species populations in biofilms with peptide nucleic acid fluorescence in situ hybridization (PNA FISH). PLoS One.

[38] Benoit MR, Conant CG, Ionescu-Zanetti C, Schwartz M, Matin A (2010). New device for high-throughput viability screening of flow biofilms. Appl. Environ. Microbiol..

[39] Roder HL, Liu W, Sorensen SJ, Madsen JS, Burmolle M (2019). Interspecies interactions reduce selection for a biofilm-optimized variant in a four-species biofilm model. Environ. Microbiol. Rep..

[40] Liu W, Russel J, Burmolle M, Sorensen SJ, Madsen JS (2018). Micro-scale intermixing: a requisite for stable and synergistic co-establishment in a four-species biofilm. ISME J..

[41] Malic S (2009). Detection and identification of specific bacteria in wound biofilms using peptide nucleic acid fluorescent in situ hybridization (PNA FISH). Microbiology.

[42] Valm AM (2011). Systems-level analysis of microbial community organization through combinatorial labeling and spectral imaging. Proc. Natl Acad. Sci. USA.

[43] Costa AM, Mergulhao FJ, Briandet R, Azevedo NF (2017). It is all about location: how to pinpoint microorganisms and their functions in multispecies biofilms. Future Microbiol..

[44] Wagner M, Nielsen PH, Loy A, Nielsen JL, Daims H (2006). Linking microbial community structure with function: fluorescence in situ hybridization-microautoradiography and isotope arrays. Curr. Opin. Biotechnol..

[45] Mattei MR (2018). Continuum and discrete approach in modeling biofilm development and structure: a review. J. Math. Biol..

[46] Borenstein DB, Meir Y, Shaevitz JW, Wingreen NS (2013). Non-local interaction via diffusible resource prevents coexistence of cooperators and cheaters in a lattice model. PLoS ONE.

[47] Bridier A, Briandet R, Bouchez T, Jabot F (2014). A model-based approach to detect interspecific interactions during biofilm development. Biofouling.

[48] Xavier JB, Martinez-Garcia E, Foster KR (2009). Social evolution of spatial patterns in bacterial biofilms: when conflict drives disorder. Am. Nat..

[49] Kreft JU, Picioreanu C, Wimpenny JW, van Loosdrecht MC (2001). Individual-based modelling of biofilms. Microbiology.

[50] Mettler E, Carpentier B (1997). Location, enumeration and identification of the microbial contamination after cleaning of EPDM gaskets introduced into a milk pasteurization line. Dairy Sci. Technol..

[51] Thomen P (2017). Bacterial biofilm under flow: first a physical struggle to stay, then a matter of breathing. PLoS One.

[52] Coyte KZ, Schluter J, Foster KR (2015). The ecology of the microbiome: networks, competition, and stability. Science.

[53] Gonze D, Coyte KZ, Lahti L, Faust K (2018). Microbial communities as dynamical systems. Curr. Opin. Microbiol..

[54] Monmeyran A (2018). The inducible chemical-genetic fluorescent marker FAST outperforms classical fluorescent proteins in the quantitative reporting of bacterial biofilm dynamics. Sci. Rep..

[55] Hynes JT (1977). Statistical mechanics of molecular motion in dense fluids. Annu. Rev. Phys. Chem..

[56] Monard C, Gantner S, Bertilsson S, Hallin S, Stenlid J (2016). Habitat generalists and specialists in microbial communities across a terrestrial-freshwater gradient. Sci. Rep..

[57] Mitri S, Clarke E, Foster KR (2016). Resource limitation drives spatial organization in microbial groups. ISME J..

[58] Karygianni L, Ren Z, Koo H, Thurnheer T (2020). Biofilm matrixome: extracellular components in structured microbial communities. Trends Microbiol..

[59] Galy O (2012). Mapping of bacterial biofilm local mechanics by magnetic microparticle actuation. Biophysical J..

[60] Mitchell KF (2015). Community participation in biofilm matrix assembly and function. Proc. Natl Acad. Sci. USA.

[61] Ben Said S, Tecon R, Borer B, Or D (2020). The engineering of spatially linked microbial consortia - potential and perspectives. Curr. Opin. Biotechnol..

[62] Nadell CD, Foster KR, Xavier JB (2010). Emergence of spatial structure in cell groups and the evolution of cooperation. PLoS Comput. Biol..

[63] Mukherjee S, Bassler BL (2019). Bacterial quorum sensing in complex and dynamically changing environments. Nat. Rev. Microbiol..

[64] An D, Danhorn T, Fuqua C, Parsek MR (2006). Quorum sensing and motility mediate interactions between Pseudomonas aeruginosa and Agrobacterium tumefaciens in biofilm cocultures. Proc. Natl Acad. Sci. USA.

[65] Moons P, Van Houdt R, Aertsen A, Vanoirbeek K, Michiels CW (2005). Quorum sensing dependent production of antimicrobial component influences establishment of E. coli in dual species biofilms with Serratia plymuthica. Commun. Agric Appl Biol. Sci..

[66] Bowen WH, Burne RA, Wu H, Koo H (2018). Oral biofilms: pathogens, matrix, and polymicrobial interactions in microenvironments. Trends Microbiol..

[67] McIntosh RPHA (1995). Gleason’s ‘individualistic concept’ and theory of animal communities: a continuing controversy. Biol. Rev. Camb. Philos. Soc..

[68] Liautaud K, van Nes EH, Barbier M, Scheffer M, Loreau M (2019). Superorganisms or loose collections of species? A unifying theory of community patterns along environmental gradients. Ecol. Lett..

[69] Nadell CD, Xavier JB, Foster KR (2009). The sociobiology of biofilms. FEMS Microbiol. Rev..

[70] Sheppard AE, Poehlein A, Rosenstiel P, Liesegang H, Schulenburg H (2013). Complete genome sequence of bacillus thuringiensis strain 407 Cry. Genom. Announc..

[71] Plamont MA (2016). Small fluorescence-activating and absorption-shifting tag for tunable protein imaging in vivo. Proc. Natl Acad. Sci. USA.

[72] Lagendijk EL, Validov S, Lamers GE, de Weert S, Bloemberg GV (2010). Genetic tools for tagging Gram-negative bacteria with mCherry for visualization in vitro and in natural habitats, biofilm and pathogenicity studies. FEMS Microbiol. Lett..

[73] Schneider RP (1996). Conditioning film-induced modification of substratum physicochemistry–analysis by contact angles. J. Colloid Interface Sci..

