# Supplementary material for: Four species of bacteria deterministically assemble to form a stable biofilm in a millifluidic channel
Source: NPJ Biofilms Microbiomes. 2021 Aug 5;7:64. doi: 10.1038/s41522-021-00233-4 (PMC8342524; doi:10.1038/s41522-021-00233-4)
Supplement: Supplementary file 7 — Supplementary Information [file 41522_2021_233_MOESM7_ESM.pdf]

Supplementary Information

Supplementary Tables

Supplementary Table 1: Media composition

Composition	**M1** (g/L)	**MB** (g/L)
Yeast Nitrogen Base1	1.7	1.7
Ammonium sulfate	5	5
Glucose	10	0.4
Casamino-acids	5	1

[1]from DIFCO BD

Supplementary Table 2: Pre-cultures

**Species**	**Exponential phase[1] OD**	**Dilutions[2]**
Bacillus thuringiensis	1.736	1 :110
Pseudomonas fluorescens	0.77	1 :360
Kocuria varians	0.67	1 :125
Rhodocyclus	0.12	1 :550

[1]Exponential phases were obtained from dilutions of overnight cultures in M1 — seeded with a single colony from M1-agar plates — grown at 30°C under agitation up to defined ODs depending on the cultured species.

[2]Dilution factor of the exponentially-growing cells to obtain 10^6 cells/mL before injection into the PDMS channel.

Supplementary figures

Supplementary Figure 1

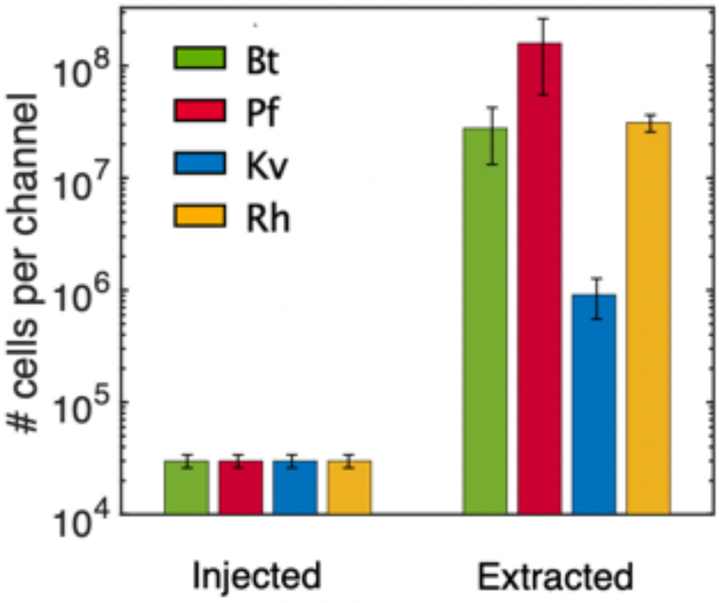

Supplementary Figure 1: Bar graph of the species counts.
Cells are injected at 10^6 cells/ml (i.e. 3.10^4/chnl) at time t=0 (left bar series), then recovered after extraction of the 4S biofilm from the channel at time t=36h. Cells are enumerated using flow cytometry and plate counting. Error bars are standard deviations of three independent determinations.

Supplementary Figure 2

A

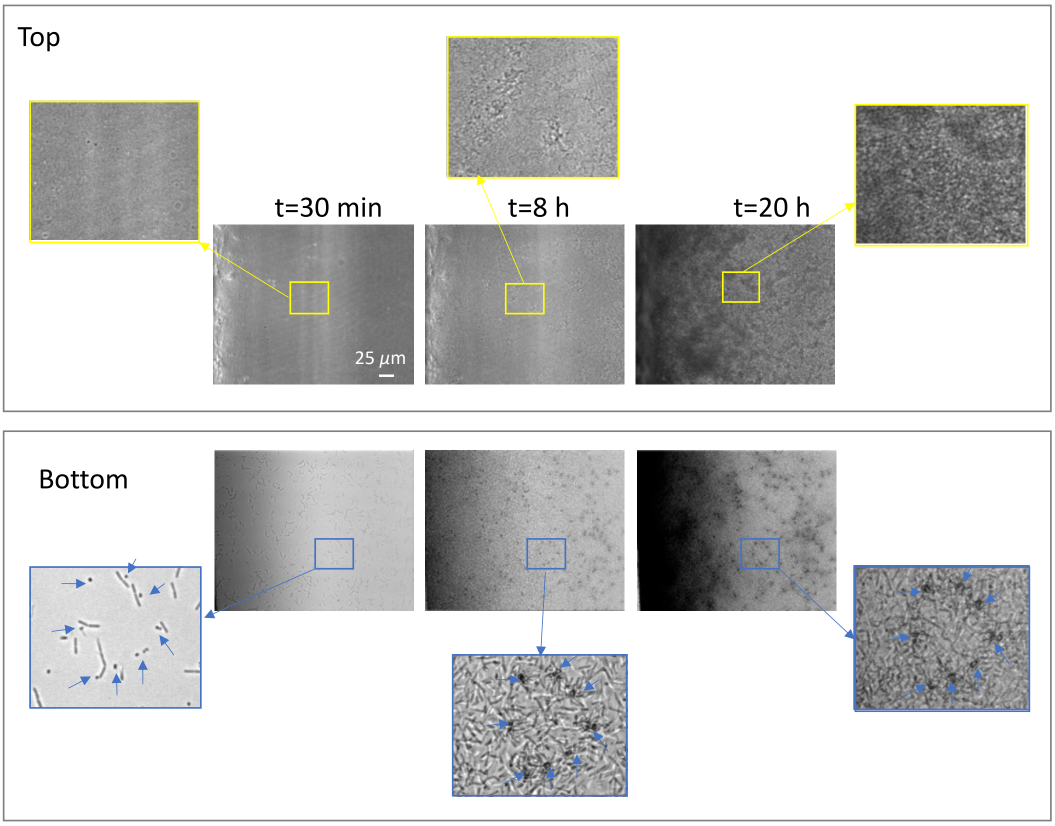

B

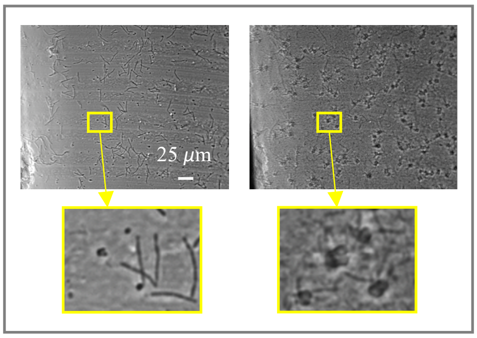

Supplementary Figure 2: *Kv* cells are present on bottom and absent on top surface. (A) Transmitted light images are taken focusing either on top surface (upper panel) or on bottom surface at time t= 30min, 8h and 20h. Inserts (yellow on top and blue on bottom) point to 4.5 x zoom in details showing *Kv* cells are present and steadily fixed (blue arrows) from the beginning on bottom surface and absent on top surface. Only *Pf* cells could be visualized after a few hours as confirmed by the fluorescence signal (see Fig. 5C of article). (B) As the optical quality of PDMS on top is lower than that of glass on bottom surface, we confirmed that *Kv* was clearly detected on top surface when present by incubating the cells in a top side down configuration of the device, i.e. PDMS down and glass up. In this configuration, the cells sediment and attach to PDMS where they can then be imaged as shown on panel B pictures. Images are acquired as usual in the upright configuration glass down and PDMS up.

Supplementary Figure 3

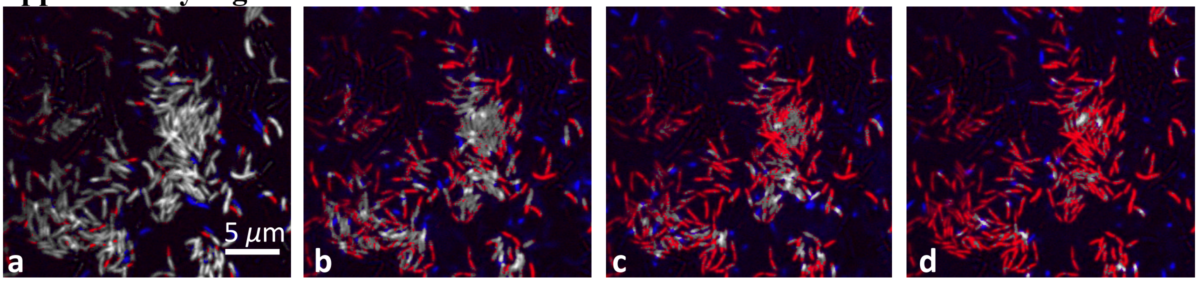

Supplementary Figure 3: *Pf* detaches in less than 2hours.
Colocalization maps of *Pf*-mCherry in the 4S community analyzed between time t =6h and time t' with t'= t +10 min (a); 40min (b); 1h (c); 1h30 (d). Light grey pixels are unchanged between t and t', blue pixels are dark at t and lighted at t'(newly appeared cells); red are lighted at t and dark at t'(removed cells).

Supplementary Figure 4

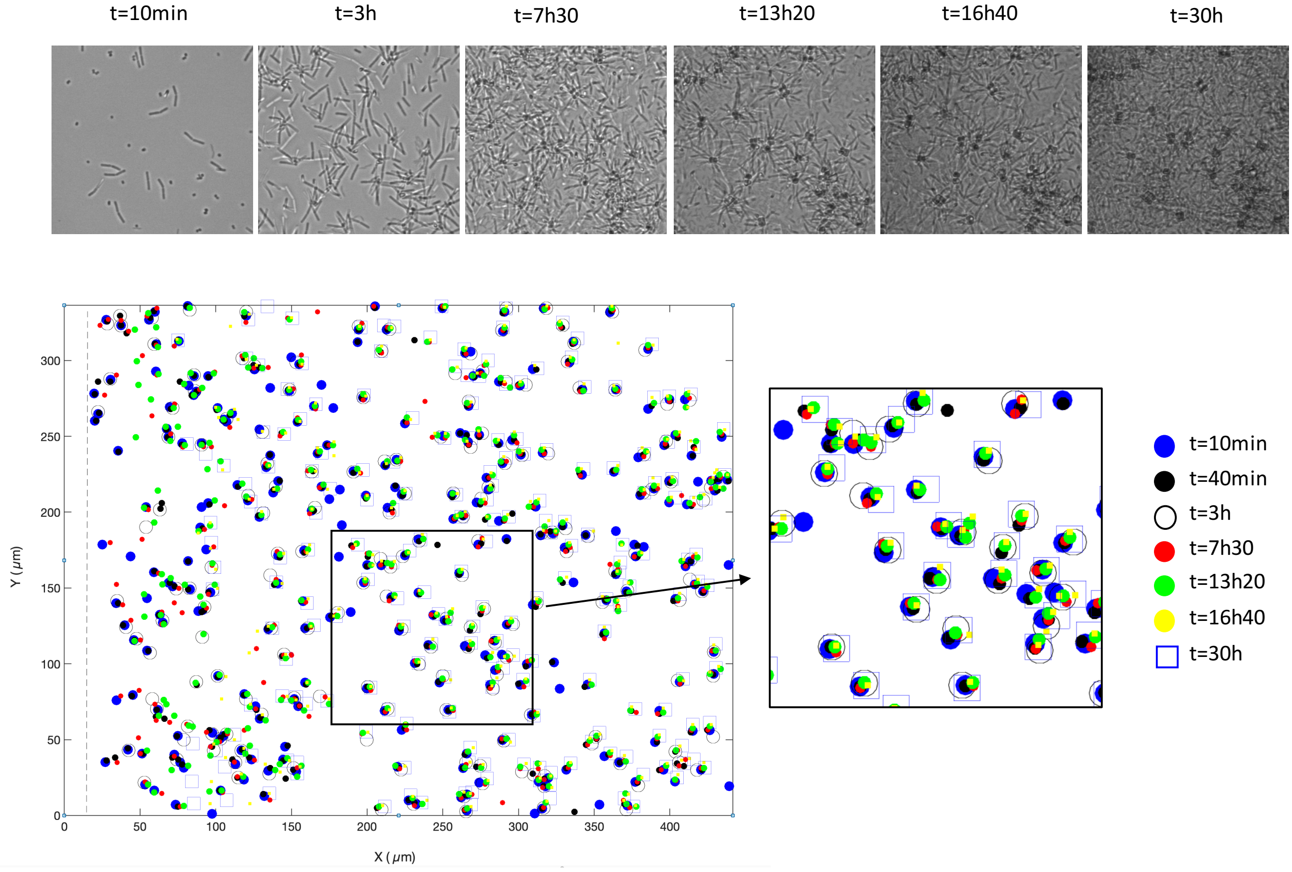

Supplementary Figure 4: *Kv* clusters spatial distribution stability along 4S biofilm development.
Overlay of *Kv* clusters (x,y) coordinates from 10 min to 30hours. The dashed line indicates the edge of the channel. As time elapses, biofilm density at the edge of the channel increases drastically and *Kv* clusters become hardly detectable accurately, the reason why almost no cluster is detected at t=30h for $x<100\mu$m. Elsewhere in the channel, more than 90% of the *Kv* clusters detected at t=30h issue from single cells stuck at the same location at time t=10 min. Yet, 15% of the single cells detected at t=10 min have detached between t=10 min and t=40min. From the map of coordinates, we calculate a formal correlation coefficient based on Pearson's coefficient definition (see Fig. S3) considering an equivalent image made of a matrix of 14336 units (actual image size divided by an *Kv* cluster size taken equal to 96 pixels) where *Kv* positions have a value (equivalent intensity) of 1 and non-*Kv* positions a value of 0. Thereby, the correlation coefficient between images displaying 90% overlap of the *Kv* positions was found equal to 0.94.

Supplementary Figure 5

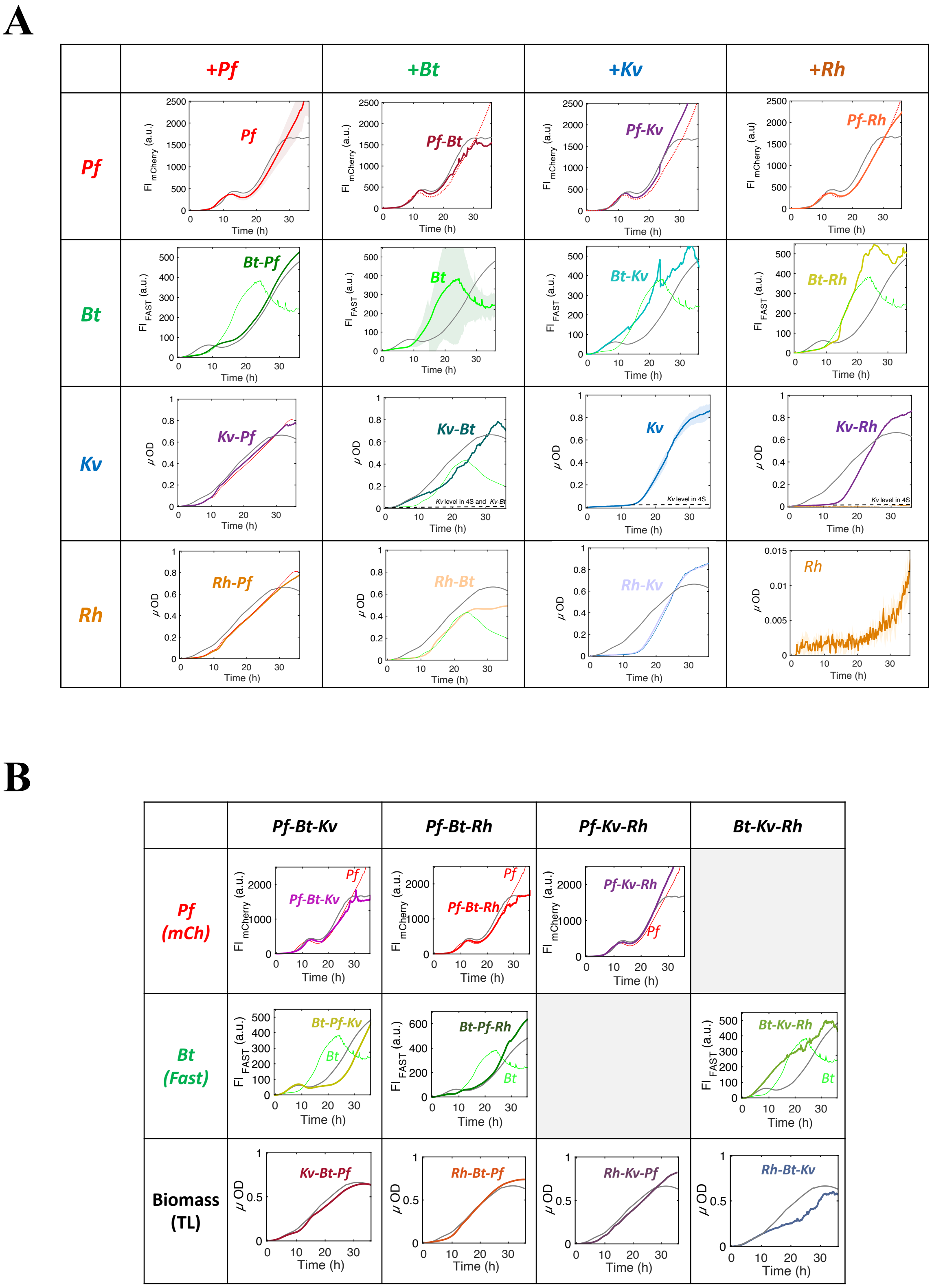

Supplementary Figure 5: Species combinatorics.
Biofilm growth kinetics are given for all the possible mixes of the 4 species. (**A**) Single-species biofilms appear in panel A in the main diagonal while off-diagonal entries show the results of pairs. In each entry, the targeted species signal — one species per line in the order *Pf*; *Bt*; *Kv*; *Rh* — is displayed in the pair formed with one of the three others — one species

par column — by a bold line. Fluorescent curves are displayed for *Pf* and *Bt* and μOD curves are shown for *Kv* and *Rh*. The corresponding signals of the targeted species in the 4S in grey are overlaid for the sake of comparison as well as the single species curves which appear as colored light lines — the targeted species in *Pf* and *Bt* lines and the paired species in *Kv* and *Rh* lines(**B**) The four triple mixes development curves are displayed in panel B — one triple mix per column — for the 3 different signals — mCherry (*Pf*), FAST (*Bt*) and TL (full biomass). The mix curve appears as a bold line, the corresponding 4S signal in grey. For fluorescent signals (lines 1 and 2) the corresponding single-species biofilm signals are displayed as a colored light line for the sake of comparison.

Supplementary Figure 6

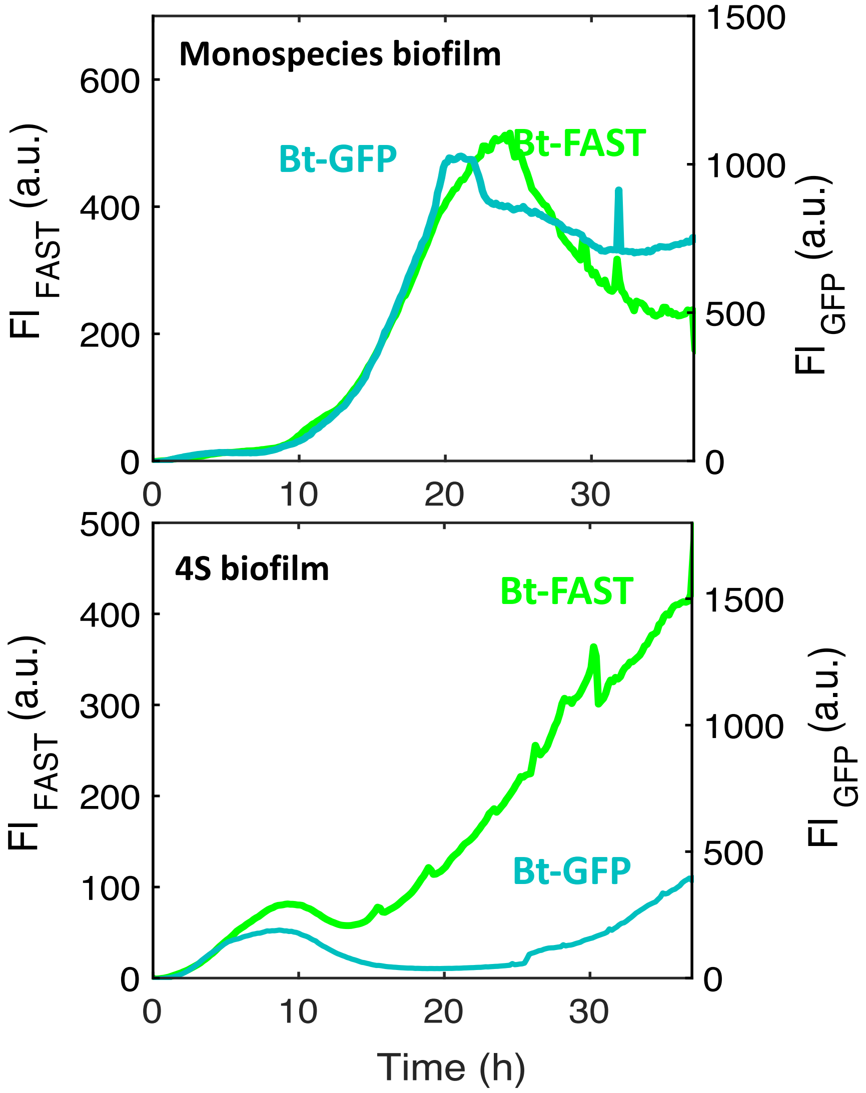

Supplementary Figure 6: Bt-FAST versus Bt-GFP in monospecies biofilm compared to 4S community.

Kinetics of the fluorescence intensity, Fl, from *Bt*-FAST and *Bt*-GFP growing in parallel channels as monospecies biofilms (upper graph) and in the 4S community (lower graph, also shown in Fig. 4 of the paper). The curves represent the average of at least three independent measurements.

Supplementary Figure 7

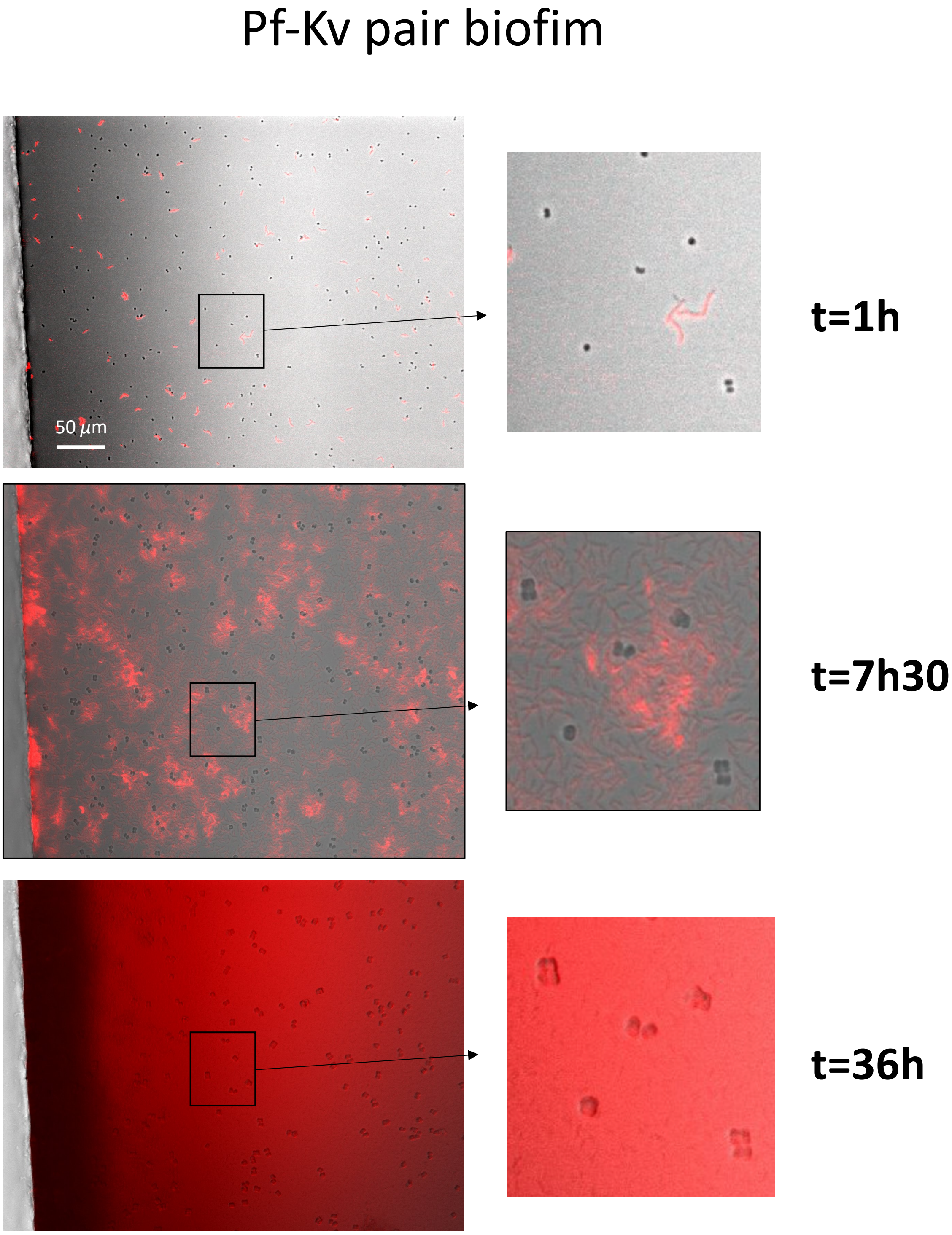

Supplementary Figure 7: *Pf* contactless inhibition of *Kv* development.
Images and zoomed-in details of the *Pf-Kv* pair biofilm from initiation to 36 hours. Fluorescence and transmitted signals are overlapped. The development of the *Kv* clusters are inhibited in the same way as for *Bt-Kv* pair or 4S biofilms although no specific physical contact can be observed between *Pf* and *Kv* cells by contrast with what is observed in the *Bt-Kv* pair.

Supplementary Figure 8

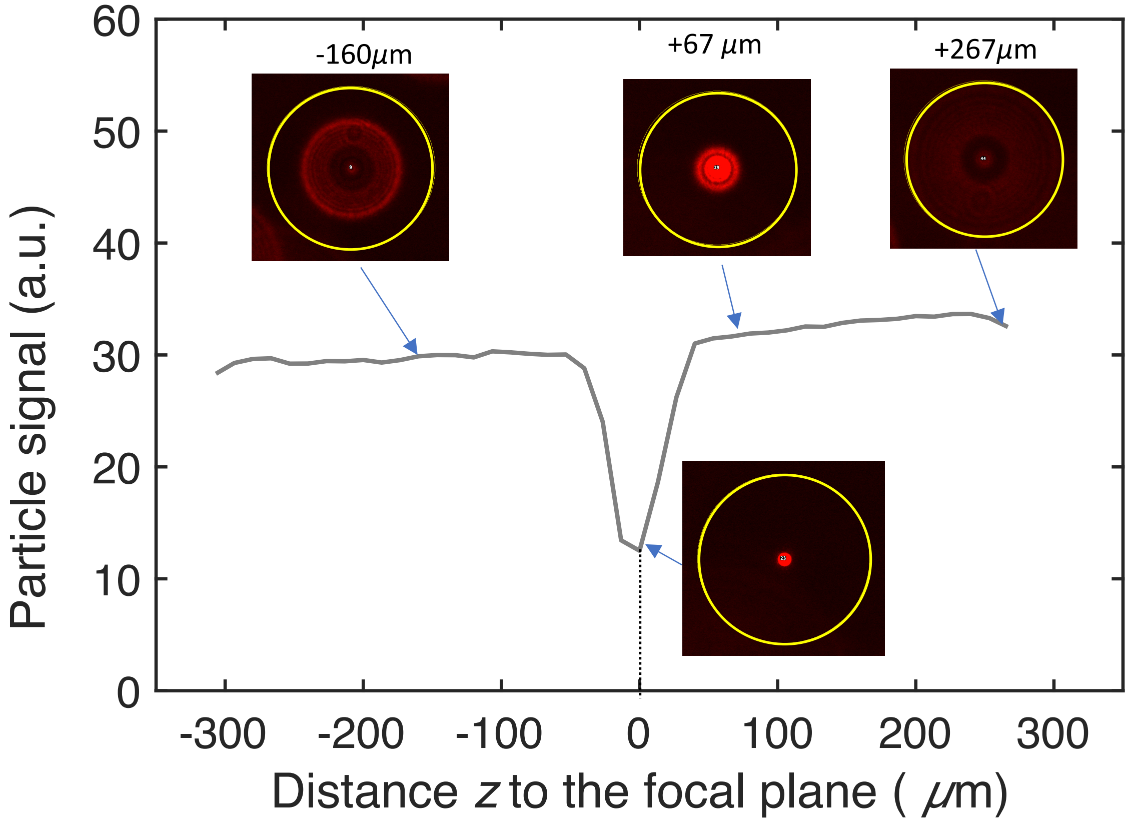

Supplementary Figure 8: **Scattered light contribution to focal plane image intensity.** An isolated fluorescent particle (1 μm in diameter), fixed in an agar-gel channel, is imaged along the optical axis (z) using a 20x objective (NA=0.45). Particle signal (pixel intensity averaged over the circular ROI delineated by the yellow line) is plotted as a function of z. The focal plane is taken as z=0. This shows the contribution of the out-of-focus objects to the focal plane and explains why *Pseudomonas fluorescens* bacteria, growing on the channel top surface contribute to the intensity of the image in-focus on the bottom surface.

Supplementary Figure 9

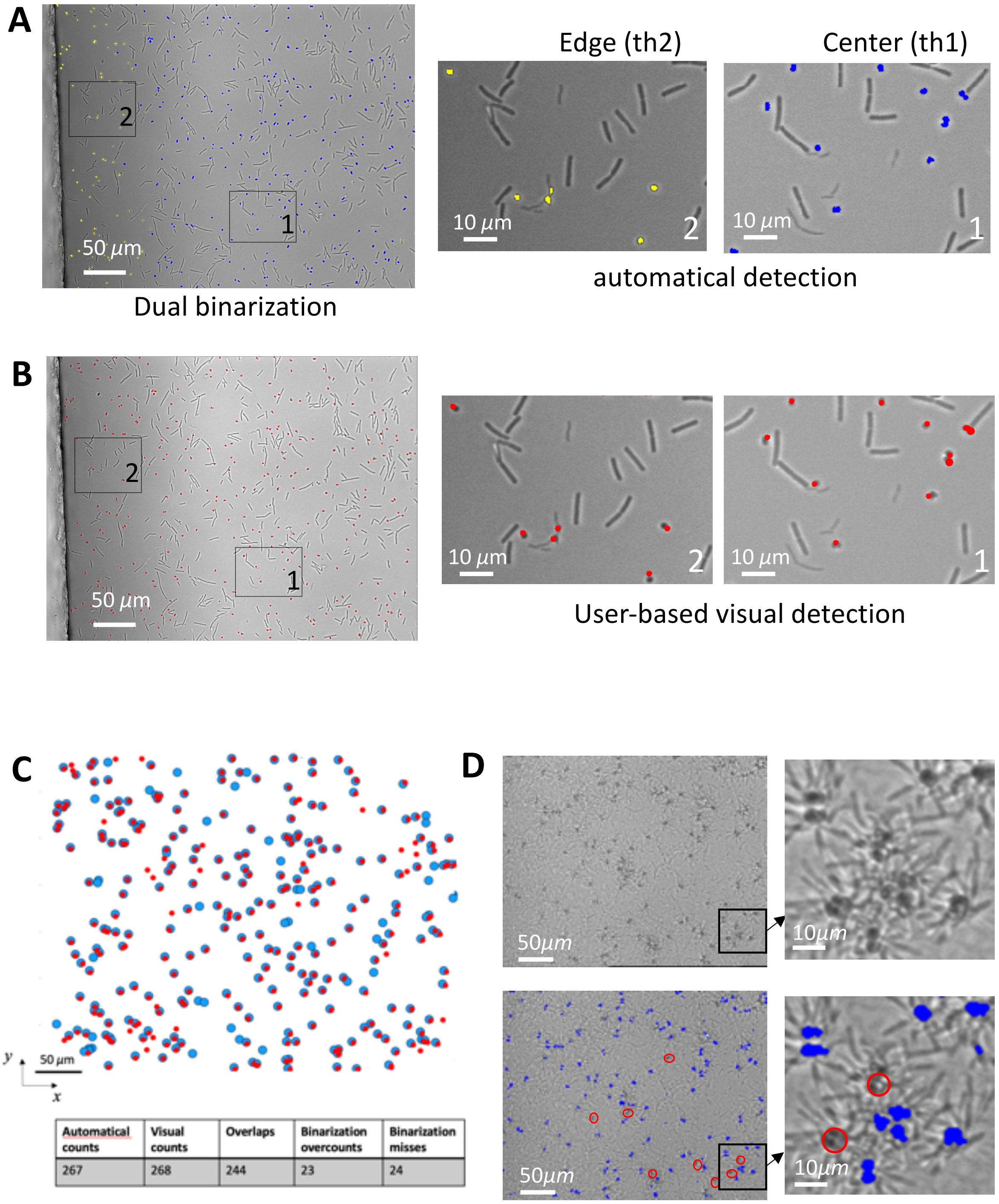

Automatical counts	Visual counts	Overlaps	Binarization overcounts	Binarization misses
267	268	244	23	24

Supplementary Figure 9: *Kv* cells and clusters detection.
(**A**) Illustration of *Kv* population detection protocol using two sets of binarization parameters; set 1 (threshold values: 392-850) for the channel center in blue and set 2 for the channel edge (threshold values: 392-635) in yellow.(**B**) User-based visual detection of *Kv* cells in red.(**C**) Overlay of automatical and user-based visual detection and comparison of the respective counts (**D**) Visually-assisted semi-automatic detection; automatic in blue visual in red.

Supplementary Figure 10

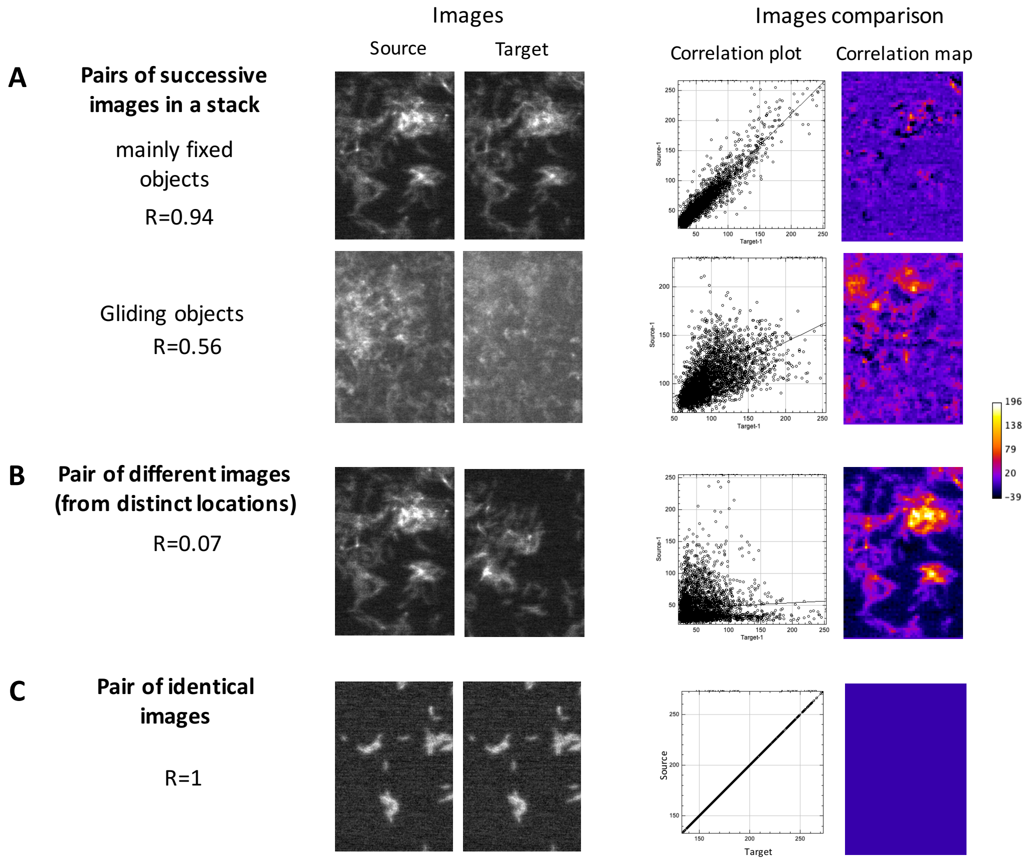

Supplementary Figure 10: Local dynamics evaluation using Pearson's coefficient.
In order to assess the local spatial dynamics of the fluorescent species within the biofilm, we carried out a correlation analysis of images by successive pairs (image n versus image n + 1). We applied the Image correlationJ * plugin whose principle is as follows: The similarity between 2 images is evaluated by calculating the Pearson correlation coefficient, R from the following equation:

$$R = \frac{\sum_{i=1}^{m}\sum_{j=1}^{n}(f(m_i,n_j)-\bar{f}\,)(g(m_i,n_j)-\bar{g}\,)}{(mn-1)\sigma_f\sigma_g}$$

where *m* and *n* are the number of pixels or group of pixels (we are using a unit region of 3 pixels side length) in the *x* and *y* directions respectively. $f\ (m_i, n_j)$ and $g\ (m_i, n_j)$ are intensity values at the position (m_i, n_j) of the image (in our case the intensity of FAST averaged over the group of pixels), $\bar{f}$ and $\bar{g}$ are the mean intensities of the entire images n and $n+1$, respectively. σ_f and σ_g are the corresponding standard deviations.

Supplementary Information
Supplementary video legends

Supplementary Video 1: 4-Species biofilm formation in a millifluidic channel
Synchronized combination of bright field (left side) and fluorescence (right side) movies of the typical development of the 4-species biofilm over 37 hours. The movie starts right after bacteria injection in the channel. Fluorescence movie shows the overlay of FAST (*Bt*) and mCherry (*Pf*) signals. The same movie is shown with two distinct fluorescence display settings. This video uses *Display1* fwhich avors visualization of mCherry signal during the first half of the biofilm formation while *display2* (Supplementary Movie 2) better exposes mCherry signal during the second half of the development. The acquisition frequency is equal to 6 frames per hour and the movie is played at 10 fps. Each field of view size is (330x440) μm^2.

Supplementary Video 2: 4-Species biofilm formation in a millifluidic channel
Same as in Supplementary Movie 1 except using *display2* which better exposes mCherry signal during the second half of the development.

Supplementary Video 3: 4-species biofilm dynamic equilibrium
This movie extract focuses on dynamic equilibrium, showing the sequence taking place in between 30 and 37 hours growth. Succession of short sequences of advancing-front progression and recession are visible.

Supplementary Video 4: Formation of *Bt*-aster around *Kv* cluster
FAST fluorescence movie showing the binding of *Bt* to *Kv* clusters after about 2 hours seeding of the 4 species in the channel. The movie starts just after injection of the bacteria in the channel and lasts 10 hours. The acquisition frequency is equal to 6 frames per hour and the movie is played at 5 fps. The field of view size is (230x253) μm^2.

Supplementary Video 5: *Bt* single-species biofilm physical instability
FAST fluorescence movie showing *Bt* single-species biofilm growth and the massive detachment that may randomly occur due to the high fragility of the biological material formed by *Bt* alone. This entrains high standard deviation of the signals averaged over at least biological replicates The acquisition frequency is equal to 6 frames per hour and the movie is played at 5 fps. The field of view size is (230x253) μm^2.

Supplementary Video 6: *Kv* single-species biofilm growth
Transmitted light movie is shown over the 36 hours growth of a *Kv* biofilm. *Kv* clusters burst dissemination and re-attachment start to be visible around image 30, i.e. time t = 15h. The acquisition frequency is equal to 2 frames per hour and the movie is played at 5 fps. Each field of view size is (330x440) μm^2.
